# Pantothenate kinase 4 controls lipid synthesis for T-cell proliferation by modulating coenzyme A and glutaminolysis

**DOI:** 10.1038/s41392-025-02385-7

**Published:** 2025-09-18

**Authors:** Jeong-Ryul Hwang, Chi Thi Ngoc Nguyen, Gwanghoon Ko, Jung-Ah Kang, Yeongseon Byeon, Seowoo Park, Ryunha Chang, Dawoon Jung, Mi Yeon Jeon, Young Hoon Sung, Cho-Rong Lee, Ki-Hoan Nam, Je Kyung Seong, Sankar Ghosh, Yun Pyo Kang, Sung-Gyoo Park

**Affiliations:** 1 College of Pharmacy and Research Institute of Pharmaceutical Science, Seoul National University, Seoul, Republic of Korea; 2 School of Life Sciences, Gwangju Institute of Science and Technology (GIST), Gwangju, Republic of Korea; 3 Bio-Nanotechnology Research Center, Korea Research Institute of Bioscience and Biotechnology, Daejeon, Republic of Korea; 4 Department of Cell and Genetic Engineering, Asan Medical Center, University of Ulsan College of Medicine, Seoul, Republic of Korea; 5 Laboratory Animal Resource and Research Center, Korea Research Institute of Bioscience and Biotechnology, Cheongju, Republic of Korea; 6 Korea Mouse Phenotyping Center, Seoul National University, Seoul, Republic of Korea; 7 Department of Microbiology and Immunology, College of Physicians and Surgeons, Columbia University, New York, NY USA

**Keywords:** Adaptive immunity, Lymphocytes

## Abstract

During T-cell-mediated inflammatory responses, T cells are activated upon recognizing specific antigens presented by antigen-presenting cells. This recognition initiates signaling through the TCR and CD28, leading to their activation and subsequent clonal expansion. Within the signaling cascades triggered by TCR and CD28 engagement, the CD28-PI3K pathway serves as a central regulator of metabolic reprogramming in T cells, supporting the biosynthetic needs essential for their effective proliferation. In this study, we found that the regulation of PANK4 plays a role in TCR/CD28-mediated CD4^+^ T-cell proliferation by regulating de novo lipid synthesis. The CD28 signaling pathway negatively regulates PANK4 through direct binding with PDK1, thereby controlling de novo lipid synthesis for CD4^+^ T-cell proliferation. Interestingly, we found that *Pank4*-deficient CD4^+^ T cells enhance coenzyme A synthesis and glutaminolysis, whereby glutamine contributes carbon for fatty acid synthesis and provides nitrogen for coenzyme A biosynthesis. The regulatory role of PANK4 in CD4^+^ T-cell proliferation was confirmed in models of experimental colitis and influenza A virus infection, where *Pank4*-deficient CD4^+^ T cells exhibited greater expansion than their wild-type counterparts when co-transferred. Our findings suggest that PANK4 regulation of de novo lipid synthesis is crucial for TCR/CD28-induced CD4^+^ T-cell proliferation and represents a potential target for modulating general CD4^+^ T-cell responses.

## Introduction

T-cell-mediated immunity is one of the major cellular defense mechanisms against pathogens. When T cells encounter antigen-presenting cells, the T-cell receptor (TCR)/CD28-mediated signaling cascade drives their activation and clonal expansion.^1^ The number of antigen-specific T cells increases through clonal expansion, enabling responses against pathogens.^2^ The proliferation of T cells can be regulated by various stimuli, with TCR/CD28 stimulation being regarded as the most essential signal for this process.^3^ Costimulation of the CD28 molecule triggers the phosphoinositide 3-kinase (PI3K) pathway, which plays important roles in regulating T-cell proliferation, survival, and death.^4^ The CD28-PI3K pathway is responsible for the metabolic reprogramming of T cells during their activation to meet the demands for robust proliferation, including energy production and macromolecule synthesis.^5^ The PI3K-AKT pathway enhances glucose transporter 1 expression and hexokinase 2 activity, thereby promoting aerobic glycolysis.^6–8^ In addition, mTORC1 regulates transcription factors involved in metabolic processes, such as peroxisome proliferator-activated receptor-γ (PPAR-γ) and sterol regulatory element-binding protein 1 and 2.^9^

Metabolites themselves regulate T-cell immunity, influencing T-cell proliferation and lineage commitment under various metabolic conditions.^5,7^ Restricting serine or glucose in the culture medium reduces T-cell proliferation,^10–12^ whereas excessive L-arginine supplementation delays proliferation and promotes differentiation into central memory-like T cells.^13^ Cytosolic aspartate, supplied by the malate-aspartate shuttle, is critical for Th1 cell proliferation.^14^ Additionally, high extracellular lactate concentrations suppress CD4^+^ T-cell proliferation.^15^ Emerging evidence highlights the role of lipid metabolic pathways in regulating T-cell functions. Inhibiting acetyl-coenzyme A (CoA) carboxylase 1 (ACC1) or PPAR-γ disrupts optimal CD4^+^ T-cell proliferation.^9^ Notably, ACC1 inhibition supports memory CD4^+^ T-cell formation by suppressing de novo fatty acid synthesis.^16^ For CD8^+^ T cells, effector CD8^+^ T cells upregulate de novo lipid synthesis to sustain TCR-mediated signaling and functions.^17^ ACC1-mediated lipid synthesis is also essential for maintaining the balance between Th17 and Treg differentiation.^18^ Although the CD28-PI3K pathway has been implicated in regulating de novo lipid synthesis, its direct role in controlling lipid metabolic pathways remains unclear.^19^

De novo lipid synthesis is a process of generating lipids from cytosolic acetyl-CoA and is regulated by various enzymes and metabolites.^20^ CoA is a vital metabolite for lipid synthesis that mediates various biochemical reactions in cells.^21^ Pantothenate kinases (PANKs) control CoA synthesis. PANK1-3 are rate-limiting enzymes that phosphorylate pantothenate (also known as vitamin B5) to initiate CoA synthesis,^22^ whereas PANK4 has been identified as a phosphatase with a pseudokinase domain.^23^ PANK4 negatively regulates CoA synthesis by dephosphorylating 4’-phosphopantetheine, an intermediate in CoA synthesis, under the regulation of the PI3K pathway.^24^ Additionally, PANK4 mediates the salvage pathway of CoA synthesis by hydrolyzing damaged forms of intermediate metabolites.^25^ Deletion of *PANK4* in both cancerous and immortalized cell lines increases CoA synthesis and promotes cell proliferation.^24^

In this study, we demonstrated that PANK4 regulation plays a pivotal role in regulating TCR/CD28-induced CD4^+^ T-cell proliferation by controlling de novo lipid synthesis. During CD4^+^ T-cell activation, PANK4 is directly regulated by the CD28-PI3K-3-phosphoinositide-dependent protein kinase 1 (PDK1) signaling pathway, which negatively modulates its activity. This regulation affects CoA synthesis and glutaminolysis, consequently regulating de novo lipid synthesis; these are essential processes for robust TCR/CD28-induced CD4⁺ T-cell proliferation. Most importantly, in vivo mouse models further supported the role of PANK4 regulation in CD4^+^ T-cell proliferation under physiological conditions. Our findings suggest that PANK4 acts as a critical intermediary between CD28 signaling and lipid metabolism during TCR/CD28-induced CD4^+^ T-cell activation.

## Results

### CD28-PI3K signaling suppresses PANK4 activity

**Fig. 1:**
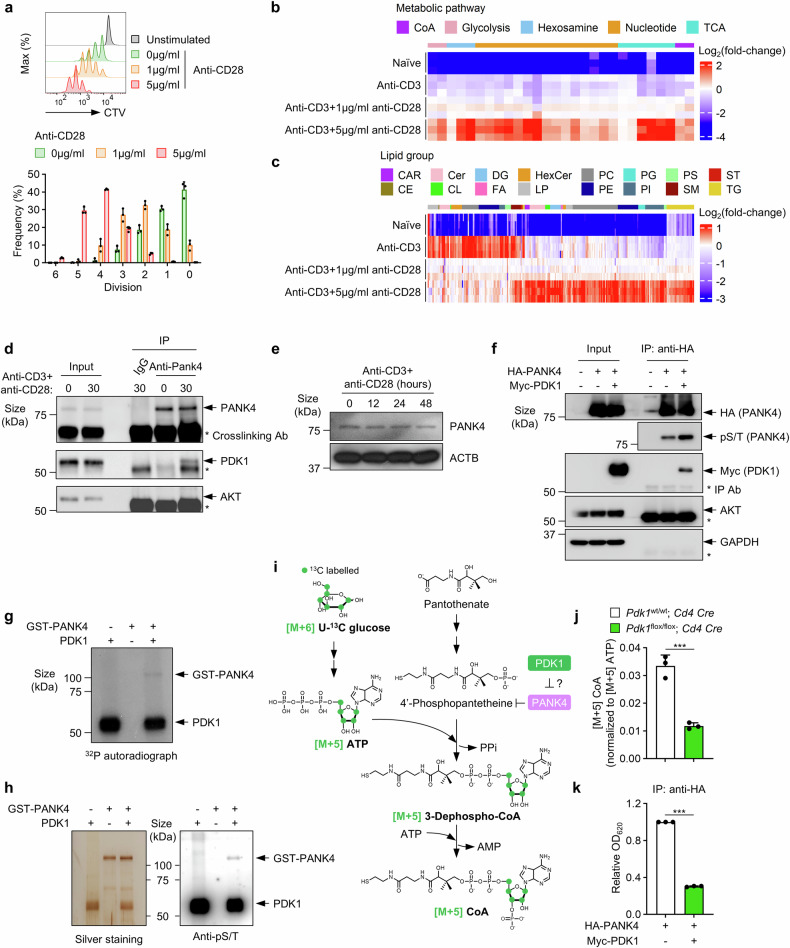
The CD28-PDK1 pathway suppresses PANK4 phosphatase activity. **a** CellTrace Violet proliferation assay of CD4^+^ T cells stimulated with 2 μg/ml anti-CD3 antibody and the indicated amount of anti-CD28 antibody for 72 h (*n* = 3). **b** Global metabolomics of naïve CD4^+^ T cells or CD4^+^ T cells stimulated for 48 h. **c** Lipidomics of naïve CD4^+^ T cells or CD4^+^ T cells stimulated for 48 h. **d** Coimmunoprecipitation using CD4^+^ T cells with or without 30 min of stimulation. **e** PANK4 expression levels after stimulating CD4^+^ T cells for the indicated durations. **f** Coimmunoprecipitation using HEK293T cells overexpressing HA-PANK4 or both HA-PANK4 and Myc-PDK1. **g** Autoradiograph of PDK1 in vitro kinase assay. **h** Silver-stained gel (left) and immunoblot of anti-phospho-S/T (right) after PDK1 in vitro kinase assay. **i** Schematic representation of the PANK4 phosphatase activity test via U-^13^C glucose tracing. **j** M + 5 coenzyme A peak area normalized to the M + 5 ATP peak area in stimulated CD4^+^ T cells (*n* = 3). **k** In vitro phosphatase assay against 4’-phosphopantetheine after immunoprecipitation of HA-PANK4 in lysates from overexpressed HEK293T cells (*n* = 3). The data are representative of two (**a**), three (**b**–**d**, **f**–**h**, **j**, and **k**), or four (**e**) independent experiments. The data are presented as the means ± SDs. Statistical analysis was performed via an unpaired two-tailed t test. *P ≤ 0.05, **P ≤ 0.01, and ***P ≤ 0.001. CAR carnitine, Cer ceramide, DG, diacylglycerol, HexCer hexosylceramide, PC phosphatidylcholine, PG phosphatidylglycerol, PS phosphatidylserine, ST sterol, CE cholesteryl ester, CL cardiolipin, FA fatty acid, LP lysophospholipid, PE phosphatidylethanolamine, PI phosphatidylinositol, SM sphingomyelin, TG triacylglycerol

The role of PDK1 in the regulation of CD4^+^ T-cell proliferation was demonstrated in a previous study.^26^ We hypothesized that there may be undiscovered proteins that interact with PDK1 or molecules downstream of PDK1 that also affect T-cell proliferation. To identify novel PDK1-binding proteins, we established Jurkat cells, a human leukemic cell line that stably expresses recombinant PDK1, via a GFP reporter coexpression system (supplementary Fig. 1a). We selected a cell clone that expressed recombinant PDK1 at low levels to minimize potential artificial effects from the overexpression (supplementary Fig. 1b). The recombinant PDK1 protein was purified via the tag pull-down method (supplementary Fig. 1c), and PDK1 and other purified proteins were visualized via silver staining (supplementary Fig. 1d). We identified one of the copurified proteins as PANK4 via mass fingerprinting (supplementary Fig. 1e, f). The binding between PDK1 and PANK4 was verified by coimmunoprecipitation (co-IP) after the PDK1 and PANK4 proteins were overexpressed in HEK293T cells (supplementary Fig. 1g). In addition, co-IP assays with truncated forms of PDK1 and PANK4 overexpressed in HEK293T cells indicated that the kinase domain of PDK1 and the fumble (pseudokinase) domain of PANK4 are crucial for their interaction (supplementary Fig. 2). CD28 stimulation is crucial for CD4^+^ T-cell proliferation. As expected, CD28 stimulation resulted in robust murine primary CD4^+^ T-cell proliferation in a dose-dependent manner (Fig. 1a; hereafter, CD4^+^ T cells refer to murine primary CD4^+^ T cells unless otherwise specified). Moreover, CD28 stimulation significantly increased the abundance of lipids, as well as metabolites, across various classes (Fig. 1b, c). These findings imply that robust proliferation of CD4^+^ T cells is accompanied by metabolic reprogramming toward anabolic metabolism. Since PANK4 is regulated by the PI3K pathway,^24^ it may be regulated by CD28 stimulation in T cells. To investigate whether the binding between PDK1 and PANK4 is dependent on TCR/CD28 signaling, we performed co-IP experiments with murine primary CD4^+^ T cells with or without TCR/CD28 stimulation. TCR/CD28 stimulation promoted the interaction between PDK1 and PANK4 (Fig. 1d). However, we were unable to detect an interaction between PANK4 and AKT through co-IP experiments (Fig. 1d), although PANK4 has been reported to be phosphorylated by AKT.^24^ We assessed PANK4 expression levels during CD4⁺ T-cell activation via an immunoblot assay, and PANK4 protein bands were quantified via densitometric analysis (supplementary Fig. 3). We stimulated CD4⁺ T cells for 12–48 h and observed no significant changes in PANK4 expression levels during this period (Fig. 1e and supplementary Fig. 3a). Given that PANK4 lacks a typical substrate motif for PDK1, we investigated the consequences of PDK1-PANK4 binding. Interestingly, the overexpression of both PDK1 and PANK4 in HEK293T cells increased the level of phosphorylated PANK4 (Fig. 1f and supplementary Fig. 3b). To verify this finding, we conducted in vitro kinase assays with purified PDK1 and purified GST-PANK4 proteins. The results from metabolic labeling with γ-^32^P ATP or an immunoblotting assay against phospho-serine/threonine revealed that purified PDK1 directly phosphorylates purified GST-PANK4 (Fig. 1g, h), as studies using GST-tagged substrates have shown that PDK1 does not phosphorylate the GST tag.^27,28^ Next, we employed stable isotope tracing with uniformly labeled (U-^13^C) glucose to assess the effect of PDK1 on PANK4 phosphatase activity by measuring the M + 5 CoA fraction (Fig. 1i). We detected a lower M + 5 CoA to M + 5 ATP ratio in *Pdk1*-deficient CD4^+^ T cells than in wild-type CD4^+^ T cells, suggesting that PANK4 phosphatase activity is increased in *Pdk1*-deficient CD4^+^ T cells (Fig. 1j and supplementary Fig. 4). Additionally, an in vitro phosphatase assay revealed that PDK1 overexpression suppressed the ability of PANK4 to degrade 4’-phosphopantetheine (Fig. 1k). These results indicate that the interaction between PDK1 and PANK4 leads to the phosphorylation of PANK4 and reduces its phosphatase activity.

### CD4^+^ T cells lacking PANK4 exhibit increased proliferation during activation

**Fig. 2:**
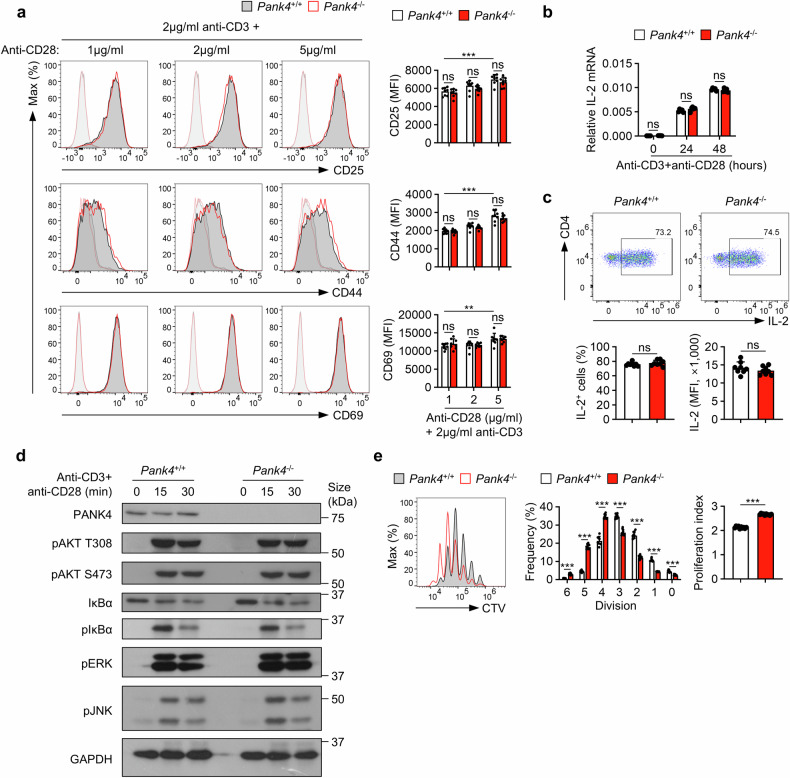
PANK4 suppresses the proliferation of CD4^+^ T cells. **a** Expression of CD4^+^ T-cell activation markers after stimulation for 24 h under the indicated conditions (*n* = 8). **b** Real-time quantitative PCR analysis of *Il-2* mRNA in stimulated CD4^+^ T cells after stimulation for the indicated durations (*n* = 5). **c** Flow cytometric analysis of intracellular IL-2 in CD4⁺ T cells stimulated with anti-CD3 and anti-CD28 antibodies for 48 h (*n* = 8). **d** Immunoblots of TCR/CD28 downstream signaling molecules after stimulation of CD4^+^ T cells for the indicated durations. **e** CellTrace Violet proliferation assay of CD4^+^ T cells stimulated with anti-CD3 and anti-CD28 antibodies for 72 h. Bar graphs represent the frequency of each peak (left) and the proliferation index (right) (*n* = 10). The data are representative of two (**c**) or three (**a**, **b**, **d**, and **e**) independent experiments. The data are presented as the means ± SDs. Statistical analysis was performed via two-way ANOVA with Tukey’s post hoc test (**a**) and the Mann‒Whitney U test (**b**, **c**, and **e**). ns not significant, *P ≤ 0.05, **P ≤ 0.01, and ***P ≤ 0.001

To investigate whether *Pank4* deficiency results in different immunological phenotypes, we generated global *Pank4*-deficient (*Pank4*^−/−^) mice (supplementary Fig. 5a, b). These mice exhibited normal CD4^+^ T-cell development (supplementary Fig. 5c, d) and did not present any significant developmental defects. Deletion of *Pank4* did not considerably affect TCR/CD28-induced activation of CD4^+^ T cells, as evidenced by negligible changes in activation marker expression at 24 h or interleukin (IL)-2 production at 24 and 48 h poststimulation (Fig. 2a-c). Moreover, CD4^+^ T-cell differentiation remained unaltered (supplementary Fig. 6). In addition, the phosphorylation levels of AKT, IκBα, ERK, and JNK, as well as IκBα degradation, were comparable between wild-type and *Pank4*-deficient CD4^+^ T cells after 15 or 30 minutes of TCR/CD28 stimulation (Fig. 2d and supplementary Fig. 3c), indicating that early TCR/CD28 signaling was not affected by the deletion of *Pank4*. However, we observed a greater proliferation rate—a 25.11% ± 0.01% increase in the proliferation index—in *Pank4*-deficient CD4^+^ T cells than in wild-type CD4^+^ T cells after 72 h of in vitro TCR/CD28 stimulation (Fig. 2e). These observations suggest that PANK4 is dispensable for activation marker expression and IL-2 production and does not affect early TCR/CD28 signaling. Therefore, we hypothesized that PANK4 may regulate CD4^+^ T-cell proliferation through another mechanism, such as modulating metabolic processes during CD4^+^ T-cell activation.

### PANK4 regulates lipid metabolism during CD4^+^ T-cell proliferation

**Fig. 3:**
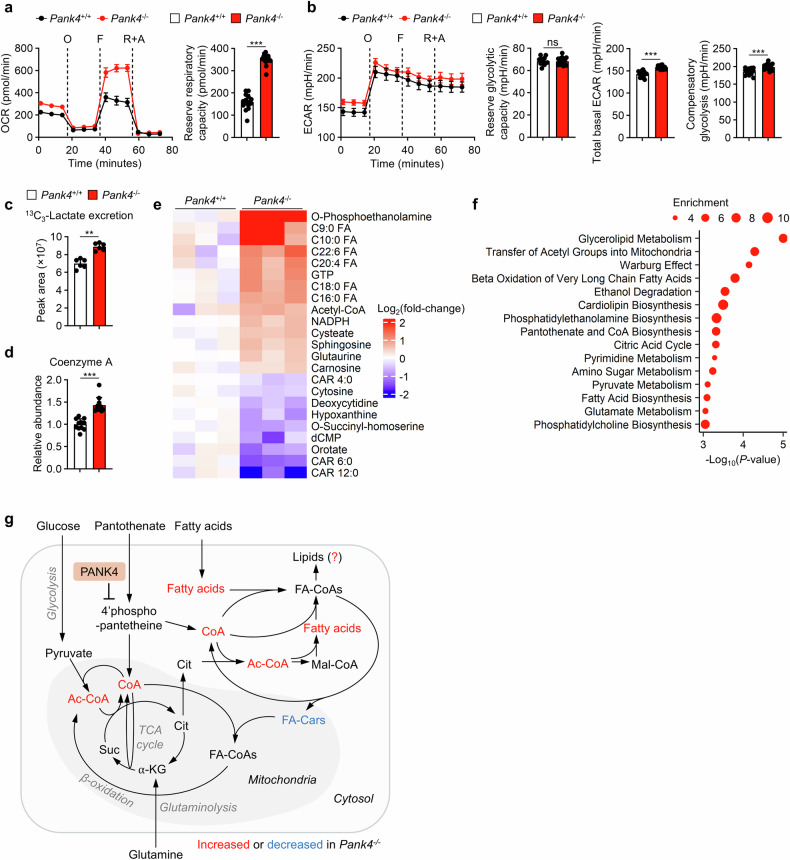
PANK4 deficiency increases the levels of metabolites related to lipid synthesis in activated CD4^+^ T cells. Seahorse assay with 48 h-stimulated CD4^+^ T cells for measuring the OCR (**a**) and ECAR (**b**) (*n* = 15). O, oligomycin; F, FCCP; R + A, rotenone + antimycin A **c** ^13^C_3_-Lactate levels in culture media after 4 h of incubation of stimulated CD4^+^ T cells in U-^13^C-glucose-supplemented media (*n* = 6). **d** Coenzyme A levels in CD4^+^ T cells stimulated with anti-CD3 and anti-CD28 antibodies for 48 h (*n* = 11). **e** Global metabolomics of CD4^+^ T cells stimulated with anti-CD3 and anti-CD28 antibodies for 48 h (*n* = 3). **f** Metabolic pathways enriched in stimulated *Pank4*^−/−^ CD4^+^ T cells. **g** Schematic representation of metabolic pathways related to differentially regulated metabolites identified through global metabolomics. The data are representative of two (**c**, **d**) or three (**a**, **b**, and **e**) independent experiments. The data are presented as the means ± SDs. Statistical analysis was performed via the Mann‒Whitney U test. *P ≤ 0.05, **P ≤ 0.01, and ***P ≤ 0.001. CoA coenzyme A, Ac-CoA acetyl coenzyme A, Cit citrate, α-KG α-ketoglutarate, Suc succinate, FA-CoA fatty acyl coenzyme A, FA-Car fatty acyl carnitine

Stimulated wild-type and *Pank4*-deficient CD4^+^ T cells were subjected to Seahorse assays to evaluate their oxygen consumption rates (OCRs) and extracellular acidification rates (ECARs) and investigate whether *Pank4*-deficient CD4^+^ T cells exhibit alterations in metabolic regulation. *Pank4*-deficient CD4^+^ T cells presented increased maximal mitochondrial respiratory capacity and significantly increased reserve respiratory capacity (Fig. 3a). Although the glycolytic reserve was unchanged, both the basal ECAR (which reflects both glycolytic and mitochondrial acidification) and compensatory glycolysis were consistently elevated (Fig. 3b). Increased glycolysis was further confirmed by measuring ¹³C₃-lactate secretion after a 4-hour incubation with U-¹³C-glucose, which was significantly greater in *Pank4*-deficient CD4^+^ T cells (Fig. 3c). Global metabolomics revealed a significant increase in CoA levels in *Pank4*-deficient CD4^+^ T cells (Fig. 3d). Additionally, we observed an accumulation of acetyl-CoA and fatty acids, along with a reduction in several carnitines (Fig. 3e). Metabolic pathway enrichment analysis of the metabolites whose abundance increased indicated that pathways related to lipid biosynthesis were enriched (Fig. 3f). Thus, we hypothesize that elevated total CoA levels may promote acetyl-CoA availability for both TCA cycle activity and de novo fatty acid synthesis. In addition, the availability of CoA can also facilitate the generation of fatty acyl CoA, which is the active form of fatty acid for incorporation into lipids (Fig. 3g).

**Fig. 4:**
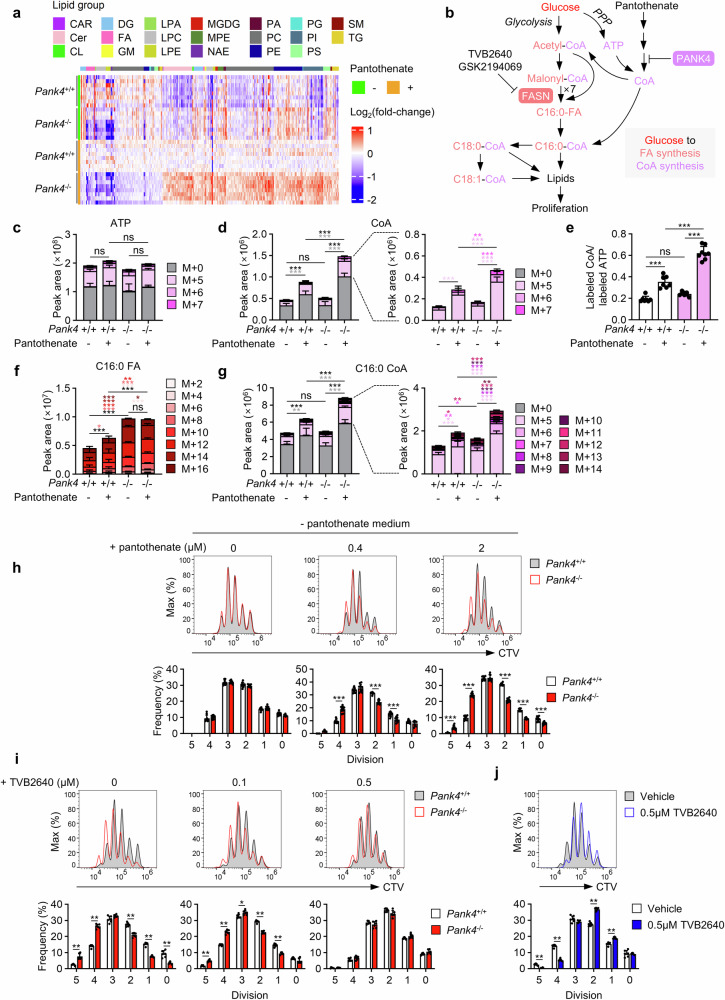
PANK4 suppresses coenzyme A synthesis, thus controlling lipid synthesis for CD4^+^ T-cell proliferation. **a** Lipidomics of CD4^+^ T cells stimulated with anti-CD3 and anti-CD28 antibodies for 48 h in media with or without 2 μM pantothenate (*n* = 8). **b** Schematic representation of the hypothesis on coenzyme A and lipid synthesis. **c–g** Quantification of metabolic intermediates in CD4^+^ T cells stimulated with anti-CD3 and anti-CD28 antibodies for 48 h in media with or without 2 μM pantothenate, followed by 4 h U-^13^C glucose tracing (n ≥ 7). The bar graphs represent the levels of ATP (**c**) and coenzyme A (**d**) and the labeled coenzyme A peak area normalized to the labeled ATP peak area (**e**) and the levels of palmitic acid (**f**) and palmitoyl coenzyme A (**g**). **h** CellTrace Violet proliferation assay of CD4^+^ T cells stimulated with anti-CD3 and anti-CD28 antibodies for 72 h in media containing the indicated amounts of pantothenate (n = 10). **i** CellTrace Violet proliferation assay of CD4^+^ T cells stimulated with anti-CD3 and anti-CD28 antibodies for 72 h in the presence of the indicated doses of TVB2640 (n = 6). **j** CellTrace Violet proliferation assay of *Pank4*^+/+^ CD4^+^ T cells stimulated with anti-CD3 and anti-CD28 antibodies for 72 h in the presence of 0.5 μM TVB2640 (n = 6). The data are representative of three independent experiments. The data are presented as the means ± SDs. Statistical analysis was performed via one-way ANOVA followed by the Bonferroni post hoc correction for (**c-g**) and the Mann‒Whitney U test for (**h**–**j**). For (**c**), statistical comparisons were conducted on the individual labeled isotopologues and the sum of all labeled isotopologues. For (**d**, **f**, and **g**), statistical analyses were performed on each labeled isotopologue (colored asterisks correspond to the respective isotopologue) and the sum of labeled isotopologues (indicated by black asterisks). ns not significant, *P ≤ 0.05, **P ≤ 0.01, and ***P ≤ 0.001. CAR carnitine, CL cardiolipin, LPA lysophosphatidic acid, LPC lysophosphatidylcholine, LPE lysophosphatidylethanolamine, FA fatty acid, Cer ceramide, DG diacylglycerol, PA phosphatidic acid, PC phosphatidylcholine, PG phosphatidylglycerol, PS phosphatidylserine, PE phosphatidyl-ethanolamine, PI, phosphatidyl-inositol, CE cholesteryl ester, SM sphingomyelin, TG triacylglycerol, NAE N-acyl ethanolamine, MGDG monogalactosyl-diacylglycerol, GM ganglioside GM1, MPE methylphosphatidyl-ethanolamine, C16:0-FA palmitate, C16:0-CoA palmitoyl CoA, C18:0-CoA, stearoyl CoA, C18:1- CoA oleoyl CoA

For detailed investigations of lipid metabolism in *Pank4*-deficient CD4^+^ T cells, we first conducted lipidomics after stimulation of the cells (supplementary Fig. 7). The results revealed that the abundance of most lipid types was increased in *Pank4*-deficient CD4^+^ T cells (supplementary Fig. 7a), which was further supported by the observation of increased neutral lipid levels in activated *Pank4*-deficient CD4^+^ T cells (supplementary Fig. 7b). To directly assess the role of CoA availability in the increased lipid content of activated *Pank4*-deficient CD4^+^ T cells, we examined lipid levels in stimulated CD4⁺ T cells under pantothenate-supplemented or pantothenate-depleted conditions, as pantothenate is a key precursor for CoA synthesis. While pantothenate supplementation led to significant lipid accumulation in *Pank4*-deficient cells compared with wild-type cells, pantothenate withdrawal markedly reduced these differences (Fig. 4a), indicating that the altered lipid phenotype in *Pank4*-deficient T cells is largely dependent on CoA biosynthesis. To compare the altered lipid phenotype with that induced by stronger CD28 stimulation (5 μg/ml compared with 1 μg/ml anti-CD28 antibody), we calculated the fold change and assessed its statistical significance. We found that more than 90% of the lipids significantly altered in *Pank4*-deficient CD4^+^ T cells under pantothenate-supplemented conditions were regulated in a manner similar to that in CD4^+^ T cells subjected to stronger CD28 stimulation (supplementary Fig. 8a), with a positive correlation coefficient of 0.67 (*P* value < 0.0001) (supplementary Fig. 8b). Furthermore, among phospholipids exhibiting significant alterations in *Pank4*-deficient CD4^+^ T cells, there was a strong negative correlation between the number of double bonds in the phospholipids and their fold change between *Pank4*-deficient and wild-type CD4^+^ T cells (supplementary Fig. 8c), where polyunsaturated lipids tended to decrease, whereas saturated and monounsaturated phospholipids increased in *Pank4*-deficient CD4^+^ T cells. Indeed, 89.3% of the phospholipids whose abundance decreased in *Pank4-*deficient CD4^+^ T cells were polyunsaturated fatty acid (PUFA)-conjugated, whereas PUFAs accounted for only 30.5% of the lipids whose abundance increased (supplementary Fig. 8d). This trend was particularly notable in the phosphatidylinositol subclass (supplementary Fig. 8d), a key lipid involved in PI3K signaling,^29^ and was also mirrored in the CD28 stimulation dataset (supplementary Fig. 8e, f). Consistent results were obtained under normal culture conditions outside of the pantothenate restriction context, and the lipid profile of *Pank4*-deficient CD4⁺ T cells closely resembled that induced by stronger CD28 stimulation. Over 90% of the altered lipids followed a similar regulatory pattern, with a positive correlation coefficient of 0.55 (*P* value < 0.0001) (supplementary Fig. 7c-d). Lipid levels were increased in *Pank4*-deficient CD4^+^ T cells, which were predominantly conjugated with saturated fatty acids (SFAs) and monounsaturated fatty acids (MUFAs) (supplementary Fig. 7c-f). This lipid signature is consistent with the hypothesis that de novo synthesized fatty acids are upregulated and actively incorporated into membrane lipids in the absence of PANK4 function.

To validate our hypothesis, we conducted U-^13^C-glucose tracing under pantothenate-supplemented and pantothenate-depleted conditions (Fig. 4b). As expected, newly synthesized CoA—represented by the M + 5 to M + 7 isotopologues derived from ^13^C-labeled ATP incorporation (Fig. 4c)—was significantly elevated in *Pank4*-deficient cells compared with wild-type CD4⁺ T cells in the presence of pantothenate. However, this increase was abolished under pantothenate-depleted conditions (Fig. 4d, e), validating the system as a model for studying endogenous CoA regulation. We then traced the labeling of palmitic acid (C16:0 FA) and palmitoyl-CoA (C16:0 CoA), key precursors for lipid biosynthesis (supplementary Fig. 9a). As expected, in wild-type cells, labeled palmitic acid was significantly greater under pantothenate-supplemented conditions than under pantothenate-depleted conditions, which was consistent with CoA availability (Fig. 4f). These findings suggest that CoA levels regulate de novo fatty acid synthesis. However, *Pank4*-deficient CD4^+^ T cells presented consistently elevated palmitic acid labeling regardless of pantothenate status (Fig. 4f). In contrast, the labeling of palmitoyl-CoA in these cells closely mirrored CoA availability—which markedly increased with pantothenate supplementation but substantially decreased with depletion (Fig. 4g and supplementary Fig. 9a). This discrepancy indicates that in the absence of sufficient CoA, the newly synthesized fatty acids cannot be efficiently converted into their active acyl-CoA form and thus accumulate, confirming that CoA deficiency limits fatty acid utilization for lipid synthesis. Moreover, a similar pattern was observed for longer-chain acyl CoAs, including stearoyl CoA (C18:0 CoA) and oleoyl CoA (C18:1 CoA), and their corresponding fatty acids (supplementary Fig. 9b-e). Specifically, significant increases in both acyl-CoA and free fatty acid contents were detected only in *Pank4*-deficient cells cultured under pantothenate-supplemented conditions, where CoA levels were elevated (Fig. 4d). In contrast, all other conditions with lower CoA availability resulted in no such increases, further supporting the conclusion that adequate CoA levels are necessary for the synthesis of newly synthesized fatty acids and their efficient conversion into their acyl-CoA forms. Together, these findings demonstrate that both fatty acid synthesis and adequate CoA availability are essential for generating fatty acyl-CoAs and supporting lipid biosynthesis in proliferating CD4⁺ T cells.

To determine whether CoA availability or fatty acid synthesis is critical for proliferation, we manipulated CoA levels via pantothenate-supplemented or pantothenate-depleted conditions and inhibited fatty acid synthesis with two potent fatty acid synthase (FASN) inhibitors, TVB-2640 and GSK2194069, during CD4⁺ T-cell activation (Fig. 4b). As expected, pantothenate depletion significantly impaired the proliferation of *Pank4*-deficient CD4⁺ T cells (Fig. 4h). Similarly, treatment with either FASN inhibitor abolished the increased proliferation observed in *Pank4*-deficient cells (Fig. 4i and supplementary Fig. 9f). At higher concentrations, both inhibitors also suppressed the proliferation of wild-type CD4⁺ T cells (Fig. 4j). Taken together, our findings highlight the synergistic role of CoA and fatty acid availability in promoting de novo lipid synthesis, thereby supporting the metabolic demands of T-cell proliferation through the regulation of PANK4 and underscoring the role of PANK4 in regulating lipid levels in CD4⁺ T-cell proliferation.

### Glutaminolysis supports lipid synthesis in *Pank4*-deficient CD4^+^ T cells

**Fig. 5:**
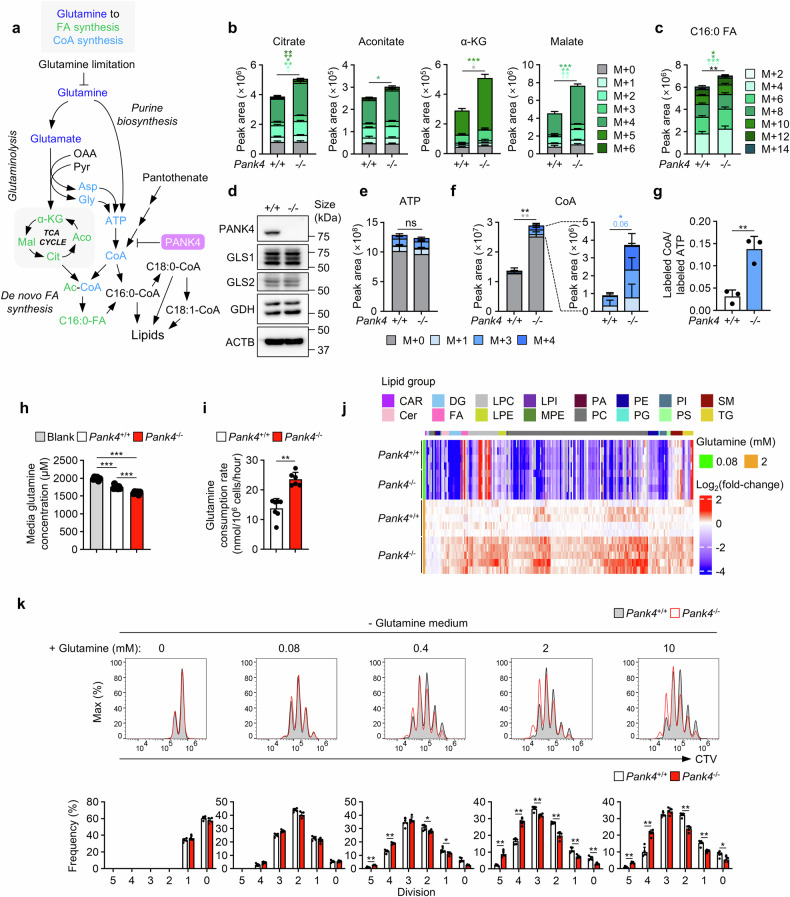
Glutamine serves as a source of both carbon and nitrogen for lipid synthesis. **a** Schematic representation of the hypothesis that glutamine supplies both carbon and nitrogen for lipid synthesis in CD4⁺ T cells. **b,** **c** Stable isotope tracing of stimulated CD4^+^ T cells using ^13^C_5_,^15^N_2_ glutamine (five carbon atoms are labeled with ¹³C, and both nitrogen atoms are labeled with ¹⁵N) for measuring TCA intermediates (*n* = 3) (**b**) and palmitate (n = 8) (**c**). **d** Expression of enzymes related to glutaminolysis after CD4^+^ T cells were stimulated for 48 hours. **e**-**g** Quantification of metabolic intermediates in CD4⁺ T cells stimulated with anti-CD3 and anti-CD28 antibodies for 48 hours, followed by tracing for 4 hours with ¹³C₅,¹⁵N₂-glutamine in media (*n* = 3). The bar graphs represent the levels of ATP (**e**) and coenzyme A (**f**) and the labeled coenzyme A peak area normalized to the labeled ATP peak area (**g**). **h** ^13^C_5_,^15^N_2_-glutamine concentration in ^13^C_5_,^15^N_2_ glutamine-containing media after 4 hours of tracing in CD4^+^ T cells stimulated for 48 hours (n ≥ 6). **i** Glutamine consumption rate of CD4^+^ T cells stimulated for 48 hours with anti-CD3 and anti-CD28 antibodies (n ≥ 6). **j** Lipidomics of CD4^+^ T cells stimulated for 48 hours with anti-CD3 and anti-CD28 antibodies in glutamine-deficient media supplemented with 0.08 mM or 2 mM glutamine (n ≥ 4). **k** CellTrace Violet proliferation assay with CD4^+^ T cells stimulated with anti-CD3 and anti-CD28 antibodies for 72 hours in glutamine-deficient media supplemented with the indicated concentrations of glutamine. The data are representative of three (**b**-**i**) or four (**d,** **j** and **k**) independent experiments. The data are presented as the means ± SDs. Statistical analysis was performed via one-way ANOVA with the Bonferroni post hoc correction (**h**), the Mann‒Whitney U test (**i** and **k**), or an unpaired two-tailed t test for all other comparisons. For (**b,** **c,** **e** and **f**), statistical comparisons were conducted on the individual labeled isotopologues (colored asterisks correspond to the respective isotopologues) and the sum of labeled isotopologues (indicated by black asterisks). ns, not significant, *P ≤ 0.05, **P ≤ 0.01, and ***P ≤ 0.001. CAR carnitine, Cer ceramide, PC phosphatidylcholine, PG phosphatidylglycerol, PS phosphatidylserine, CE cholesteryl ester, CL cardiolipin, FA fatty acid, LPC lysophosphatidylcholine, LPE lysophosphatidylethanolamine, LPI lysophosphatidylinositol, PE phosphatidyl-ethanolamine, PI phosphatidylinositol, SM sphingomyelin, TG triacylglycerol, DG diacylglycerol, NAE N-acyl ethanolamine, PA phosphatidic acid, MPE methylphosphatidyl-ethanolamine, Asp aspartate, Gly glycine, α-KG α-ketoglutarate, Aco aconitate, Mal malate, Cit citrate, Ac-CoA acetyl coenzyme A, C16:0-FA palmitate, C16:0-CoA palmitoyl CoA, C18:0-CoA, stearoyl CoA, C18:1-CoA oleoyl CoA

Given the well-established role of glutamine as a major source of both carbon and nitrogen during T-cell activation,^29–31^ we conducted ^13^C_5_,^15^N_2_ glutamine tracing in both wild-type and *Pank4*-deficient CD4^+^ T cells to dissect how PANK4 influences de novo fatty acid and CoA synthesis. We hypothesized that glutamine-derived carbon supports the TCA cycle via glutaminolysis, whereas nitrogen contributes to purine biosynthesis and ultimately ATP production, a critical precursor for CoA synthesis (Fig. 5a and supplementary Fig. 10a). Both glutamine and glutamate displayed high levels of labeling, confirming robust uptake and incorporation (supplementary Fig. 10b). As expected, the tracing results revealed that glutaminolysis was increased in *Pank4*-deficient CD4^+^ T cells (Fig. 5b), leading to increased carbon influx into de novo fatty acid synthesis (Fig. 5c), despite the unchanged expression of glutaminolysis-related enzymes (Fig. 5d and supplementary Fig. 3d). With respect to nitrogen utilization, we observed comparable ^15^N labeling of ATP in both genotypes (Fig. 5e). Interestingly, the fraction of ^15^N-labeled CoA (derived from ATP) was significantly elevated in *Pank4*-deficient cells (Fig. 5f, g), suggesting increased nitrogen flux from glutamine to CoA biosynthesis. Taken together, glutamine provides carbon for de novo fatty acid synthesis and nitrogen for CoA biosynthesis, thereby enabling the incorporation of fatty acids into complex lipids. Furthermore, we observed a significantly greater reduction in extracellular glutamine after 4 hours of incubation in *Pank4*-deficient cells (Fig. 5h), which was consistent with a higher glutamine consumption rate (Fig. 5i), implicating PANK4 in the regulation of glutamine metabolism. To further investigate the contribution of CoA availability to this phenotype, we repeated the ^13^C_5_,^15^N_2_ glutamine tracing experiments under pantothenate-supplemented and pantothenate-depleted conditions to monitor endogenous CoA levels. As expected, depletion of pantothenate was sufficient to decrease the glutaminolysis-mediated TCA cycle (supplementary Fig. 10c). ATP levels and labeling remained unaffected (supplementary Fig. 10d), moreover, the increased ^15^N labeling of CoA in *Pank4*-deficient cells under supplemented conditions was abolished by pantothenate withdrawal (supplementary Fig. 10e, f). Additionally, glutamine uptake closely correlated with CoA availability (supplementary Fig. 10g, h), suggesting that CoA modulates glutamine utilization in T cells.

To validate the role of glutamine in lipid biosynthesis, we performed lipidomic analysis under glutamine-limited conditions. As expected, the lipid accumulation observed in *Pank4*-deficient CD4⁺ T cells compared with wild-type cells under glutamine-supplemented conditions was no longer evident when glutamine was nearly depleted (Fig. 5j). Additionally, there were comparable proliferation rates between wild-type and *Pank4*-deficient CD4^+^ T cells when stimulated in low-glutamine media (below 0.08 mM), whereas *Pank4*-deficient CD4^+^ T cells exhibited enhanced proliferation with sufficient glutamine (Fig. 5k). Together, these findings demonstrate that glutamine serves as a dual-source metabolite, providing carbon for fatty acid synthesis and nitrogen for CoA biosynthesis. Both arms are essential for supporting lipid accumulation and the proliferative capacity of CD4⁺ T cells, particularly in the absence of PANK4. Thus, PANK4 acts as a critical regulator that limits glutamine-driven anabolism during T-cell activation.

### PANK4 regulates immune responses by controlling the expansion of CD4^+^ T cells in vivo

**Fig. 6:**
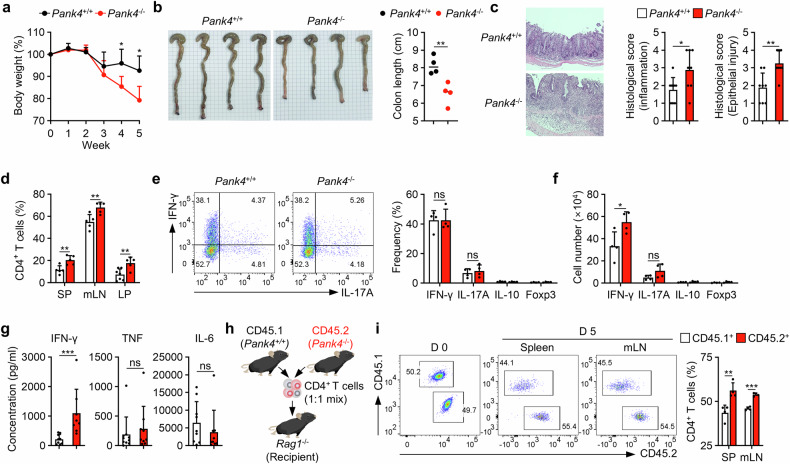
Robust proliferation of *Pank4*-deficient CD4^+^ T cells leads to severe symptoms of experimental colitis. **a** Body weight curve of *Rag1*^−/−^ mice after adoptive transfer of naïve CD4^+^ T cells (n = 4). **b** Colon length (*n* = 4). **c** Histological analyses of colon tissues (*n* = 8). **d** Proportions of CD4^+^ T cells in the indicated tissues (*n* ≥ 5). **e** Proportions of T helper cell subsets in mesenteric lymph nodes (*n* = 4). **f** Numbers of T helper cell subsets in mesenteric lymph nodes (*n* = 4). **g** Quantification of cytokine levels in the explant culture supernatants of colon tissues after 24 h (n ≥ 8). **h** Schematic representation of homeostatic proliferation in CD45.1 (*Pank4*^+/+^) and CD45.2 (*Pank4*^−/−^) mice. **i** Proportion of CD45.1^+^ and CD45.2^+^ CD4^+^ T cells after 5 days of homeostatic proliferation (*n* = 4). The data for (**a**, **b**, **e**, **f**, and **i**) are representative of three independent experiments. The data are presented as the means ± SDs. Statistical analysis was performed via the Mann‒Whitney U test for (**g**) and an unpaired two-tailed t test for all other comparisons. ns not significant, *P ≤ 0.05, **P ≤ 0.01, and ***P ≤ 0.001. SP spleen, mLN mesenteric lymph node, LP lamina propria

We investigated whether *Pank4* deficiency in CD4^+^ T cells can induce enhanced immune responses in vivo. We transferred naïve (CD25^-^CD45RB^hi^) CD4^+^ T cells into recombination activating 1 (*Rag1*)-deficient mice to induce experimental colitis by causing lymphopenia-induced naïve CD4^+^ T-cell proliferation.^32^ Compared with those administered wild-type CD4^+^ T cells, *Rag1*-deficient mice administered *Pank4*-deficient CD4^+^ T cells presented more severe colitis symptoms at the same time points (Fig. 6a-c). We observed increased proportions of CD4^+^ T cells in the spleens, mesenteric lymph nodes (mLNs), and lamina propria of the mice (Fig. 6d). As expected, the degree of differentiation of adoptively transferred CD4^+^ T cells was comparable between *Pank4*-deficient and wild-type CD4^+^ T cells (Fig. 6e). However, *Rag1*-deficient mice that received *Pank4*-deficient CD4^+^ T cells presented increased numbers of interferon (IFN)-γ-producing CD4^+^ T cells since the total number of CD4^+^ T cells was greater (Fig. 6f). Increased numbers of IFN-γ-producing CD4^+^ T cells led to increased production of IFN-γ in colon tissues, whereas tumor necrosis factor (TNF)-α or IL-6 levels did not change (Fig. 6g). To compare proliferation in vivo, naïve CD4^+^ T cells from CD45.2-expressing *Pank4*-deficient mice and CD45.1-expressing wild-type mice were mixed at the same ratio and adoptively transferred into *Rag1*-deficient mice (Fig. 6h). Five days after adoptive transfer, we observed increased ratios of *Pank4*-deficient CD4^+^ T cells (CD45.2^+^) compared with those of wild-type cells (Fig. 6i). These data demonstrate that *Pank4*-deficient CD4^+^ T cells induce enhanced immune responses because of their increased expansion rate in vivo.

### The increased expansion rate of *Pank4*-deficient CD4^+^ T cells reinforces immune protection against influenza A virus infection

**Fig. 7:**
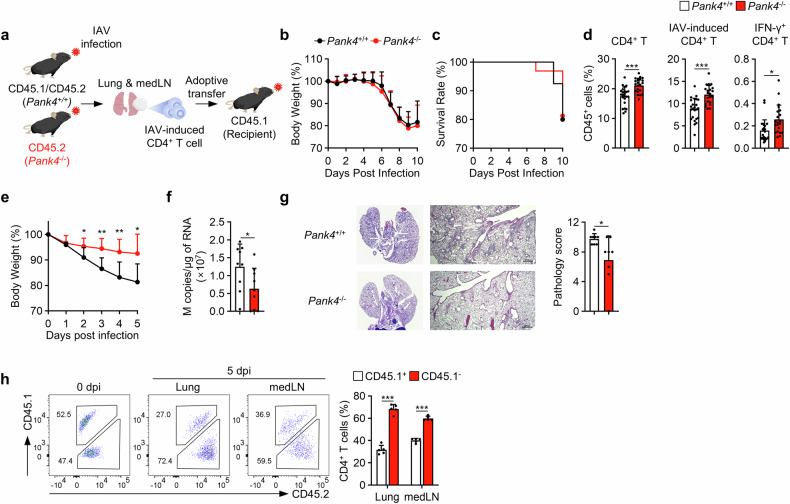
*Pank4*-deficient CD4^+^ T cells provide increased protection against influenza A virus infection. **a** Schematic representation of the assessment of IAV-induced CD4^+^ T-cell responses in primed mice infected with IAV. **b** Body weight curves of *Pank4*^+/+^ (n = 40) and *Pank4*^*−/−*^ (*n* = 32) mice with sublethal IAV infection (0.5 MLD_50_). **c** Survival curves. **d** Proportions of CD4^+^ T cells, IAV-induced CD4^+^ T cells, and IFN-γ^+^ CD4^+^ T cells among CD45^+^ cells in the lungs of *Pank4*^+/+^ (*n* = 24) and *Pank4*^−/−^ (*n* = 21) mice at 10 days postinfection after sublethal infection. **e** Body weight curves of recipient mice (*n* = 8) adoptively transferred with IAV-induced *Pank4*^+/+^ and *Pank4*^−/−^ CD4^+^ T cells following infection with a lethal dose of IAV (10 MLD_50_). **f** Viral titers in the lungs at 10 days postinfection determined by measuring the copy numbers of the M gene via real-time quantitative PCR (*n* = 9). **g** Histological analyses of the lungs of *Pank4*^+/+^ (*n* = 8) and *Pank4*^−/−^ (*n* = 7) mice at 5 days post infection after infection with a lethal dose. **h** Proportion of CD45.1^+^CD45.2^+^ (*Pank4*^+/+^) and CD45.1^-^CD45.2^+^ (*Pank4*^−/−^) CD4^+^ T cells in recipient mice that received both cell types (*n* = 5). The data in (**h**) are representative of three independent experiments. The data are presented as the means ± SDs for (**b** and **d**-**h**). Statistical analysis was performed via an unpaired two-tailed t test. *P ≤ 0.05, **P ≤ 0.01, and ***P ≤ 0.001. IAV influenza A virus, medLN mediastinal lymph node

We further hypothesized that the increased proliferation of *Pank4*-deficient CD4^+^ T cells could play an improved protective role in a mouse model of virus infection (Fig. 7a). Mice with wild-type and *Pank4*-deficient T cells were infected with a sublethal dose (0.5 MLD_50_) of influenza A virus (IAV) (supplementary Fig. 11). The body weights and survival rates of the two groups of mice were comparable at 10 days post infection (Fig. 7b, c). However, the numbers of total CD4^+^ T cells, IAV-induced (CD49d^+^ CD11a^hi^)^33^ CD4^+^ T cells and IFN-γ^+^ CD4^+^ T cells were greater in the lungs of the mice with *Pank4*-deficient T cells than in those of the mice with wild-type T cells (Fig. 7d). To exclude putative extrinsic effects of PANK4 on CD4^+^ T-cell expansion due to other cells,^34,35^ IAV-induced CD4^+^ T cells were adoptively transferred into recipient CD45.1 mice with distinct congenic markers, elucidating the specific role of *Pank4*-deficient CD4^+^ T cells in protection against IAV infection. After intravenous adoptive transfer of IAV-induced wild-type or *Pank4*-deficient CD4^+^ T cells, the recipient mice were infected with a lethal dose of IAV (10 MLD_50_). Infected recipient mice that received wild-type CD4^+^ T cells presented a rapid decrease in body weight, whereas those that received *Pank4*-deficient CD4^+^ T cells presented ameliorated body weight loss (Fig. 7e). Moreover, the adoptive transfer of *Pank4*-deficient CD4^+^ T cells reduced the IAV titer in the lungs (Fig. 7f), as did the lung inflammation and pathology scores, compared with the transfer of wild-type CD4^+^ T cells (Fig. 7g). To clarify whether the number of *Pank4*-deficient CD4^+^ T cells increases during IAV infection, we cotransferred equal numbers of wild-type and *Pank4*-deficient IAV-induced CD4^+^ T cells into mice distinguished by different congenic markers (Fig. 7a). The results revealed greater numbers of *Pank4*-deficient (CD45.1^-^CD45.2^+^) CD4^+^ T cells in both the lungs and mediastinal lymph nodes 5 days post infection than did wild-type (CD45.1^+^CD45.2^+^) CD4^+^ T cells (Fig. 7h). Our findings suggest that the increased expansion rate of *Pank4*-deficient CD4^+^ T cells strengthens immune protection against influenza A virus infection.

## Discussion

In this study, we demonstrated that direct regulation of PANK4 is crucial for TCR/CD28-induced CD4^+^ T-cell proliferation. Genetic deletion of *Pank4* in murine CD4^+^ T cells led to increased proliferation, and metabolomic and lipidomic analyses revealed that de novo lipid synthesis is essential for robust proliferation. Additionally, PDK1 is regulated primarily by CD28^26^ and directly binds to PANK4 during TCR/CD28-induced CD4^+^ T-cell activation. Coexpression of PDK1 with PANK4 reduced PANK4 activity, whereas genetic deletion of *Pdk1* increased PANK4 activity. Thus, our findings suggest that PANK4 is negatively regulated by the CD28-PDK1 signaling pathway, which promotes activated T-cell proliferation. The role of PANK4 in cell proliferation has been previously suggested.^24^ In that study, the functional role of PANK4 in CoA synthesis was identified via a cell line-based experimental system. However, the importance of PANK4 in CoA synthesis under physiological conditions has remained unclear. In this study, we directly demonstrated the critical role of PANK4 regulation in CD4^+^ T-cell responses, particularly its involvement in the proliferation of activated normal primary CD4^+^ T cells and in genetic model-based inflammatory systems. Under in vivo inflammatory conditions, lineage commitment was not significantly regulated by PANK4 deficiency; however, CD4^+^ T-cell proliferation was markedly increased in the absence of PANK4. On the basis of our findings, we propose that PANK4 regulates T-cell proliferation by modulating metabolic processes associated with de novo lipid synthesis. Furthermore, PANK4 could serve as a target for regulating T-cell responses under inflammatory conditions. Specifically, during viral infections, targeting PANK4 may enhance T-cell responses.

Previous studies have shown that the mTOR and PPAR-γ pathways associated with CD28-PI3K signaling influence lipid synthesis through the regulation of lipid synthesis-related gene expression. Additionally, these pathways impact lipid uptake and storage through gene expression control. Our data revealed that PANK4 deficiency did not differentially regulate the expression of lipid synthesis-related genes, despite an observed increase in de novo lipid synthesis. These results suggest that the CD28-PI3K pathway regulates de novo lipid synthesis via both gene expression-independent (PDK1-PANK4) and gene expression-dependent (AKT-mTOR) mechanisms. Notably, our findings highlight differences in upstream regulators and the effects of PANK4 on cell proliferation. As reported by Dibble et al.,^24^ increased proliferation was observed in cancer cell lines when PANK4 was regulated by AKT through the expression of a constitutively active AKT mutant. However, in our study, PANK4 coimmunoprecipitated with PDK1, but not with AKT, in activated CD4^+^ T cells, indicating that PDK1 regulates PANK4 activity. In addition, PDK1 can directly bind to PANK4 and phosphorylate PANK4. Interestingly, a separate study using lens epithelial cell lines reported enhanced proliferation with PANK4 overexpression, contrary to our findings.^36^ These discrepancies suggest that the role of PANK4 may be cell type-specific or context dependent, although its regulation by PDK1 promotes increased proliferation of activated CD4^+^ T cells via de novo lipid synthesis.

A recent study revealed that phosphoinositide acyl chain saturation plays a crucial role in CD8^+^ effector T-cell activation.^17^ Importantly, we also demonstrated that PANK4 inhibits de novo fatty acid synthesis and the formation of corresponding acyl-CoAs, thereby preventing the accumulation of phospholipids containing SFAs and MUFAs (supplementary Figs. 7e, f and 8c, d), which are derived primarily from de novo-synthesized lipids. The CD28-PI3K pathway inhibits PANK4 activity, leading to the accumulation of saturated and monounsaturated phospholipids. Therefore, our study suggests that CD28 signaling activates the PI3K pathway not only through PI3K phosphorylation but also through phosphoinositide acyl chain saturation through PANK4.

Glutamine serves as a pivotal nutrient for activated T cells, supplying both carbon and nitrogen for essential biosynthetic processes. While its involvement in TCA cycle replenishment via glutaminolysis and nucleotide synthesis is well established,^29–31^ its contribution to CoA biosynthesis, particularly as a nitrogen donor, has received less attention and remains underexplored. In our study, *Pank4*-deficient activated CD4^+^ T cells presented increased glutaminolysis and glutamine uptake, replenishing the TCA cycle and supporting fatty acid synthesis. Furthermore, restricting pantothenate in the culture medium reduced glutaminolysis-derived TCA cycle intermediates in *Pank4*-deficient CD4^+^ T cells, suggesting that increased CoA levels promote glutaminolysis-derived TCA cycling during T-cell activation. Importantly, our findings also revealed that glutamine-derived nitrogen contributes to CoA biosynthesis, a critical cofactor in lipid metabolism. The correlation between CoA levels and glutamine consumption in *Pank4*-deficient CD4^+^ T cells implies a regulatory mechanism, although the direct pathways involved remain to be elucidated. Collectively, our data suggest that glutamine has dual functions in activated CD4^+^ T cells: supplying carbon for energy production and biosynthesis and providing nitrogen for the synthesis of essential cofactors such as CoA. Understanding the interplay between glutamine metabolism and CoA synthesis could offer new insights into T-cell function and potential therapeutic targets. Despite these metabolic changes, differentiation into T helper subsets was not significantly affected by PANK4 deficiency, either in vitro or in vivo. In contrast, a previous study demonstrated that extracellular treatment with high concentrations of CoA promotes Tc22 or Th22 differentiation.^37^ Another study reported that high-level pantothenate treatment significantly increases the intracellular CoA level, resulting in attenuated Th17 differentiation.^38^ These discrepancies may be attributable to extrinsic factors, such as the availability of resources for CoA synthesis. Collectively, our findings imply that PANK4 influences T-cell proliferation primarily under normal conditions. However, external factors affecting CoA synthesis, such as dietary pantothenate availability, may impact T-cell differentiation.

In summary, our study demonstrated that PANK4 is directly regulated by the CD28-PI3K pathway to control the lipid metabolism essential for CD4^+^ T-cell proliferation. Metabolomic and lipidomic analyses of *Pank4*-deficient CD4^+^ T cells revealed that de novo lipid synthesis is crucial for their proliferation, which is supported by increased CoA synthesis and increased glutaminolysis. These findings deepen our understanding of the connection between metabolic regulation and CD4^+^ T-cell proliferation. Additionally, we propose that PANK4 could be a promising target for enhancing T-cell immunity and immunotherapies by controlling T-cell expansion while minimally affecting differentiation into helper subsets.

## Materials and methods

### Mice

*Pank4*^−/−^ mice were generated by the Korea Mouse Phenotyping Center (KMPC) through CRISPR-Cas9-mediated deletion of the 9th exon of the *Pank4* locus in mice on a C57BL/6 background. *Rag1*^−/−^ mice and CD45.1 mice were obtained from the Jackson Laboratory (Bar Harbor, ME, USA). *Pdk1*^flox/flox^; *Cd4 Cre* mice were generated as previously described.^26^ All the mice were maintained in a specific pathogen-free facility. Eight- to twelve-week-old, sex-matched mice were used for all the experiments. All animal experiments were approved by the Seoul National University’s Institutional Animal Care and Use Committee (SNU-230320-4) and were performed in accordance with relevant guidelines and regulations.

### Primary CD4^+^ T-cell isolation and cell culture

Murine primary CD4^+^ T cells were isolated from the spleen and peripheral lymph nodes via a naïve CD4^+^ T-cell isolation kit (130-104-453, Miltenyi Biotec, Bergisch Gladbach, Germany) according to the manufacturer’s instructions. The cells were cultured in RPMI-1640 medium supplemented with 2 mM L-glutamine, 10% FBS, 100 U/ml penicillin‒streptomycin, 10 mM HEPES, 1 mM sodium pyruvate and 50 μM β-mercaptoethanol. For in vitro stimulation, CD4^+^ T cells were seeded on plates coated with 2 μg/ml anti-mouse CD3e and 1 μg/ml anti-mouse CD28 antibodies for 12–72 h. CD4^+^ T cells were crosslinked with anti-hamster rabbit IgG following incubation with soluble anti-CD3e and anti-CD28 antibodies for 15–30 min. All the cells were cultured in a humidified incubator at 37 °C with 5% CO_2_.

### Immunoprecipitation

Primary CD4^+^ T cells or HEK293T cells were lysed by nondenaturing lysis buffer containing 20 mM Tris, 150 mM NaCl, 2 mM EDTA, 1% Triton X-100, 10% glycerol, protease inhibitors, and phosphatase inhibitors. Anti-PANK4 (12055, Cell Signaling Technology, Danvers, MA, USA) or anti-HA (3724, Cell Signaling Technology) antibodies were incubated with the lysates at a ratio of 1:50 at 4 °C overnight with gentle rotation. Protein G Sepharose 4 Fast Flow beads (17061801, Cytiva, Marlborough, MA, USA) were added to the mixture, which was subsequently incubated at 4 °C for 4 h. The beads were washed with lysis buffer and boiled in 2× Laemmli sample buffer, and the supernatants were subjected to immunoblotting.

### PDK1 in vitro kinase assay

The PDK1 in vitro kinase assay was performed as previously described^39^ with slight modifications. Purified PDK1 protein (14-452, Merck Millipore, Darmstadt, Germany) and purified GST-PANK4 protein (H00055229-P01, Abnova, Taipei, Taiwan) were incubated in kinase reaction buffer containing 50 mM Tris-Cl (pH 7.5), 10 mM MgCl_2_, 1 mM β-mercaptoethanol, 0.1 mM ATP, 10 μM PI(3,4,5)P_3_, 100 μM DOPS, and 100 μM DOPC at 30 °C for 30 min. The samples were boiled after the addition of Laemmli sample buffer and subjected to SDS‒PAGE, followed by immunoblotting or autoradiography. For autoradiography, 10 μCi of [γ-^32^P]-ATP was included in the reaction buffer.

### Seahorse assay

The OCR and ECAR were determined via Agilent Seahorse XFp and XFe96 analyzers with a Seahorse XF Cell Mito Stress Test Kit according to the manufacturer’s instructions. Briefly, 1 × 10^5^ activated CD4^+^ T cells were resuspended in XF assay medium supplemented with 10 mM glucose, 2 mM glutamine, and 1 mM pyruvate. The cells were seeded on XF96 or XFp microplates coated with poly-D-lysine and allowed to adhere to the bottom of the wells by centrifugation at 200 × *g* for 1 min without braking. After incubation in a humidified non-CO_2_ incubator at 37 °C for 30 min, the plate was loaded into the Seahorse XFp or XFe96 machine. Finally, 1.5 μM oligomycin, 1 μM carbonyl cyanide-4-(trifluoromethoxy)phenylhydrazone (FCCP), and 0.5 μM rotenone/antimycin were administered.

### T-cell transfer-induced colitis

CD4^+^CD25^-^CD45RB^hi^ T cells were isolated from the spleens and peripheral lymph nodes of the mice via a FACSAria III instrument. A total of 5 × 10^5^ cells were adoptively transferred into *Rag1*^−/−^ mice via intravenous injection into the retro-orbital sinus. Changes in body weight and stools were monitored every week to track colitis symptoms. Spleens, mesenteric lymph nodes, and colon tissues were extracted for immunological and histological analyses.

### Lamina propria cell isolation

Colon tissues were washed and cut into small pieces. The tissues were then incubated in buffer containing 1× HBSS, 5 mM EDTA, and 1 mM DTT with shaking to remove intraepithelial lymphocytes. The tissues were subsequently incubated in digestion buffer containing 0.5 mg/ml DNase I, 0.5 mg/ml collagenase D, and 3 mg/ml dispase II, with shaking at 37 °C for 20 min, twice. Single-cell suspensions were obtained by passing the digested tissues through a 70 μm mesh filter. Lymphocytes were then enriched via Percoll gradient centrifugation.

### Influenza A virus infection

Seasonal human A/H1N1 (A/Victoria/2570/2019, VI19) viruses were propagated in MDCK cells and quantified via a plaque assay with MDCK cells. Five- to seven-week-old mice were infected intranasally with 500 PFU (0.5 MLD_50_) of IAV for primary infection. After the adoptive transfer of IAV-induced CD4^+^ T cells into CD45.1 mice, the mice were infected with 10,000 PFU (10 MLD_50_) of IAV to induce aggravated pathogenicity. The viral titers in the lungs of infected mice were quantified via RNA extracted from excised lung lobes. Reverse transcribed viral RNA was measured via real-time quantitative PCR.

### Lung cell isolation

At 10 days post infection, lungs from infected mice were extracted for isolation of lymphocytes. Briefly, the lungs were digested in gentleMACS C-tubes with digestion buffer containing 0.5 mg/ml collagenase D, 0.5 mg/ml DNase, and 2 mg/ml dispase II for 20 min at 37 °C with gentle shaking. After digestion, single-cell suspensions were obtained by passing through a 70 μm mesh filter. Then, the lung lymphocytes were isolated via density gradient centrifugation. For the isolation of IAV-induced CD4^+^ T cells from the lungs and mediastinal lymph nodes of infected mice, CD45.2^+^CD3e^+^CD4^+^CD11a^hi^ cells were isolated via a FACSAria III instrument.

### Histology and scoring

Colon and lung tissues were fixed in 4% PFA and embedded in paraffin. Four-micron-thick sections were cut and stained with hematoxylin and eosin. Colon histological scores were graded on the basis of inflammation and epithelial injury, as previously described.^40^ Lung histological scores were graded on the basis of peribronchiolar inflammation and alveolitis (0=normal, 1=very mild, 2=mild, 3=moderate, 4=marked, 5=severe), as well as epithelial damage and pulmonary edema (0=normal, 1=mild, 2=moderate, 3=marked, 4=severe).

### Statistical analysis

The data are presented as the means ± standard deviations (SDs). The significance of differences between groups was assessed via the Mann‒Whitney U test, two-tailed t test, or ANOVA followed by a post hoc test. Normality was tested prior to the use of t tests or ANOVA. The statistical analysis methods used for each figure are described in the figure legends. *P* values < 0.05 were considered significant. All the statistical tests were performed via GraphPad Prism software version 10 (San Diego, CA, USA).

Other methods are described in the Supplementary Information.

## Supplementary information

Supplementary information

Uncropped blot and gel

Gating strategy

## Data Availability

All the data generated or analyzed during this study are included in this published article and its supplementary information.
