# Supplementary material for: Pantothenate kinase 4 controls lipid synthesis for T-cell proliferation by modulating coenzyme A and glutaminolysis
Source: Signal Transduct Target Ther. 2025 Sep 18;10:302. doi: 10.1038/s41392-025-02385-7 (PMC12443970; doi:10.1038/s41392-025-02385-7)
Supplement: Supplementary file 1 — Supplementary information [file 41392_2025_2385_MOESM1_ESM.docx]

Supplementary Materials for

Pantothenate kinase 4 controls lipid synthesis for T-cell proliferation by modulating coenzyme A and glutaminolysis

Jeong-Ryul Hwang^1,2^†, Chi Thi Ngoc Nguyen^1^†, Gwanghoon Ko^1^†, Jung-Ah Kang^3^, Yeongseon Byeon^2^, Seowoo Park^1^, Ryunha Chang^1^, Dawoon Jung^1^, Mi Yeon Jeon^4^, Young Hoon Sung^4^, Cho-Rong Lee^1^, Ki-Hoan Nam^5^, Je Kyung Seong^6^, Sankar Ghosh^7^, Yun Pyo Kang^1^*,
and Sung-Gyoo Park^1^*

Correspondence to: [yunpyo.kang@snu.ac.kr](mailto:yunpyo.kang@snu.ac.kr) (YPK) and [riceo2@snu.ac.kr](mailto:riceo2@snu.ac.kr) (SGP)

**This PDF file includes:**

Materials and Methods

Figures. S1 to S11

Table S1

**Other Supplementary Materials for this manuscript include the following:**

WB raw data.

Materials and Methods

Transfection

HEK293T cells were cultured in high-glucose DMEM supplemented with 10% FBS and 100 U/ml penicillin‒streptomycin in a humidified incubator at 37°C with 5% CO_2_. The cells were transfected with FugeneHD transfection reagent (E2311, Promega, Madison, WI, USA) according to the manufacturer’s instructions and incubated for 48 hours.

Immunoblot assay

The cells were lysed in Laemmli sample buffer and boiled for 10 min. The samples were subjected to SDS‒PAGE and wet transferred at 4°C overnight. The membrane was blocked with 5% nonfat dry milk in TBST and incubated with primary antibody, followed by incubation with an HRP-conjugated secondary antibody. The images were obtained by scanning the X-ray film or using an ImageQuant 800 (Cytiva).

PANK4 *in vitro* phosphatase assay

The PANK4 *in vitro* phosphatase assay was performed according to previous studies^1,2^. HEK293T cells were transfected with the pcDNA3-HA-PANK4 and pcDNA3-Myc-PDK1 plasmids and subjected to immunoprecipitation after 48 hours of incubation. After immunoprecipitation with an anti-HA antibody, the beads were incubated with 2 mM 4’-phosphopantetheine in reaction buffer containing 50 mM Tris-Cl (pH 7.5), 100 mM NaCl, and 0.5 mM Co^2+^ at 30°C for 30 min. Free phosphate in the supernatant was detected using a malachite green phosphate detection kit (DY996, R&D Systems, Minneapolis, MN, USA).

IL-2 expression analysis by real-time quantitative PCR and intracellular IL-2 staining

For real-time quantitative PCR, stimulated primary CD4^+^ T-cell pellets were lysed, and RNA was extracted via the RNeasy Mini Kit (Qiagen, Hilden, Germany). One microgram of RNA was used to synthesize cDNA, and 1–2 μl of cDNA was used to measure *Il-2* mRNA expression. For flow cytometric analysis of intracellular IL-2, primary CD4^+^ T cells were stimulated for 48 hours with anti-CD3 and anti-CD28 antibodies followed by a 4-hour treatment with 100 ng/ml phorbol 12-myristate 13-acetate (PMA), 500 ng/ml ionomycin, and 1× brefeldin A solution (00-4506-51, eBioscience, San Diego, CA, USA) at 37°C. After surface staining, the samples were incubated with the intracellular antigens for 30 min at room temperature, followed by fixation and permeabilization. The samples were analyzed via CytoFLEX (Beckman Coulter, Brea, CA, USA).

*In vitro* T helper subset polarization

Isolated murine naïve CD4^+^ T cells were differentiated into T helper subsets using the CellXVivo Mouse Th1 Cell Differentiation Kit (CDK018, R&D Systems), CellXVivo Mouse Th17 Cell Differentiation Kit (CDK019, R&D Systems), and CellXVivo Mouse Treg Cell Differentiation Kit (CDK007, R&D Systems) according to the manufacturer’s instructions. Briefly, the cells were resuspended in polarizing medium and plated on a TCR- or TCR/CD28-stimulatory antibody-coated plate. After 96 hours of incubation, intracellular cytokines were analyzed by flow cytometry.

Flow cytometry

Cell surface antigen staining was performed in 2% FBS/PBS for 20 min at room temperature, followed by staining with the Zombie Aqua Fixable Viability Kit (423102, BioLegend, San Diego, CA, USA). Fc receptors were blocked as needed with a mouse BD Fc block (553141, Becton Dickinson, Franklin Lakes, NJ, USA). For intracellular cytokine staining, cells were stimulated with 100 ng/ml phorbol 12-myristate 13-acetate (PMA), 500 ng/ml ionomycin, and 1× brefeldin A solution (00-4506-51, eBioscience) at 37°C for 4 hours. Intracellular antigens were stained for 30 min at room temperature after surface staining, followed by fixation and permeabilization. To assess the proliferation of the cells, the cells were stained using a CellTrace Violet Cell Proliferation Kit (C34557, Thermo Fisher Scientific). BODIPY 493/503 (D3922, Thermo Fisher Scientific) was used at a final concentration of 200 ng/ml to stain lipids. The samples were analyzed using FACSCanto II (Becton Dickinson), FACSLSR Fortessa (Becton Dickinson), and CytoFLEX (Beckman Coulter) systems. Detailed information on antibodies is provided in the Supplementary Information (supplementary Table 1).

LC‒MS analysis for metabolomics

For nontargeted metabolomics and lactate measurement, previously established LC‒MS conditions were employed^3^. The mobile phases used for chromatography consisted of 10 mM ammonium carbonate with 0.05% ammonium hydroxide in water (mobile phase A) and 100% acetonitrile (mobile phase B). Five microliters of sample were injected into a SeQuant ZIC-pHILIC LC column (100×2.1 mm, 5 μm, 200 Å) coupled with a SeQuant ZIC-pHILIC guard column (20×2.1 mm). The column temperature was maintained at 30°C, and the elution was performed at a flow rate of 0.25 ml/min with the following gradient: 0–13 min, 80% to 20% B; 13–15 min, 20% B; 15–15.1 min, 20% to 80% B; followed by a 5-minute postrun at 80% B. For palmitic acid and palmitoyl coenzyme A analysis, 5 µl of sample was injected into an Acquity UPLC BEH C18 column (50×2.1 mm, 1.7 μm) coupled with an Acquity UPLC BEH C18 1.7 μm VanGuard precolumn (5×2.1 mm). The column temperature was set to 30°C, and elution occurred at a flow rate of 0.3 ml/min with the following gradient: 0–0.2 min, 5% B; 0.2–1 min, 5–45% B; 1–3 min, 45% to 65% B; 3–3.2 min, 65% to 95% B; and 3.2–5 min, 95% B, followed by a 3-minute postrun at 5% B.

Mass spectrometry analysis was conducted via a Q Exactive Plus instrument (Thermo Fisher Scientific) equipped with a heated electrospray ionization (HESI) source. MS1 scans were performed in polarity switching mode for nontargeted metabolomics or in negative mode for fatty acid analysis with the following settings: sheath gas at 50 arbitrary units, auxiliary gas at 25 arbitrary units, sweep gas at 1 arbitrary unit, spray voltage at 3 kV, capillary temperature at 320°C, and auxiliary gas heater temperature at 350°C. The mass range was set to 70–1050 m/z for hydrophilic conditions and 230–550 m/z for palmitic acid and palmitoyl coenzyme A, with a resolution of 70,000 and an AGC target of 1×10^6^.

Metabolomic data analysis was performed via EI Maven version 0.12.0, with metabolite identification on the basis of retention times and m/z values from an in-house library. For nontargeted metabolomics, LC‒MS peaks were automatically extracted and aligned via the automated feature detection function of EL-Maven, followed by statistical analysis.

LC‒MS analysis for lipidomics

The samples were added to a Waters Acquity UPLC CSH C18 column (100×2.1 mm; 1.7 μm) coupled to an Acquity UPLC CSH C18 VanGuard precolumn (5×2.1 mm; 1.7 μm). Lipidomics was conducted with an injection volume of 5 µl. Lipids were separated by gradient elution using solvent A (acetonitrile/water, 60:40, v/v) and solvent B (isopropanol/acetonitrile, 90:10, v/v) containing 10 mM ammonium acetate for negative mode and 10 mM ammonium formate for positive mode. Separation was carried out at 65°C with a flow rate of 0.6 ml/min under the following gradient: 0–2 min, 15% to 30% B; 2–2.5 min, 30% to 48% B; 2.5–11 min, 48% to 82% B; 11–11.5 min, 99% B; and 11.5–12 min, 99% B, followed by a 4-minute postrun at 15% B.

MS analysis was performed on a Thermo Scientific Q Exactive Plus Quadrupole-Orbitrap instrument (Thermo Fisher Scientific) equipped with a HESI source and operated in negative or positive ion mode with the following parameters: sheath gas, 60 arbitrary units; auxiliary gas, 25 arbitrary units; sweep gas, 2 arbitrary units; spray voltage, 3 kV; capillary temperature, 320 °C; S-lens RF level, 50%; and aux gas heater temperature, 370 °C. Data-dependent acquisition (DDA) mode analysis was performed for quantification at the MS1 mass range of 200-2000, a resolution of 70,000 at m/z 200, an AGC target of 1×10^6^, and a maximum injection time of 100 ms. For MS/MS acquisition, the parameters were as follows: resolution of 17,500 at m/z 200, AGC target of 1×10^5^, maximum injection time of 50 ms, top N set to 4, isolation window of m/z 1.0, and normalized stepped collision energy of 20-30-40. Lipid peaks were identified, aligned, and exported via MS-DIAL software, and only lipids fully identified by MS2 spectra were included in the analysis.

LC-QqQ-MS based glutamine quantification

For the quantification of glutamine, an Agilent 6460 triple-quadrupole mass spectrometer connected to an Agilent 1260 HPLC system was utilized. Chromatographic separation was performed on a SeQuant ZIC-pHILIC LC column (100×2.1 mm, 5 μm, 200 Å) coupled with a SeQuant ZIC-pHILIC guard column (20×2.1 mm). The mobile phases consisted of 10 mM ammonium carbonate with 0.05% ammonium hydroxide in water (mobile phase A) and 100% acetonitrile (mobile phase B). The elution was performed at a flow rate of 0.25 ml/min with the following gradient: 0–7 min, 80% to 47% B; 7.1–10 min, 20% B; 10–10.1 min, 20% to 80% B; followed by a 4-minute post run at 80% B. The column temperature was maintained at 30°C, and the sample injection volume was set to 5 µl. The electrospray ionization (ESI) source operated in positive mode, with a gas temperature of 325°C and a sheath gas temperature of 350°C, both at a flow rate of 11 L/min. The nebulizer gas pressure was set to 35 psig, and the capillary voltage was 2750 V. The mass spectrometer was operated in multiple reaction monitoring (MRM) mode, targeting specific transitions: glutamine: m/z 147.1 ([M+H]^+^)→130.0; ^13^C_5_,^15^N_2_-glutamine: m/z 154.1([M+H]^+^)→136.0. The fragmentor voltage and collision energy were set to 60 V and 4 V, respectively. The autosampler was maintained at 4°C throughout the analysis. Data acquisition and quantification of peak areas were performed using Agilent MassHunter Qualitative Analysis B.8.0 software. The absolute concentrations of glutamine were quantified using the isotope dilution method, a highly precise approach that corrects variability in sample preparation and instrumental analysis^4^. Statistical analyses were conducted using a two-tailed unpaired Student’s t-test.

Metabolite set enrichment analysis

Pathway enrichment scores were calculated via the enrichment analysis tool in MetaboAnalyst (<https://www.metaboanalyst.ca>). Metabolites whose abundance was significantly increased in *Pank4­*^-/-^ CD4^+^ T cells compared with *Pank4­*^+/+^ CD4^+^ T cells were used for the analysis.

Glucose or glutamine, pantothenate free RPMI-1640 media preparation for isotope tracing

To enable nutrient-specific isotope tracing, custom RPMI-1640 base media lacking glucose or glutamine, and pantothenate were prepared by reconstituting individual components according to the standard RPMI-1640 formulation. Inorganic salts were first weighed and dissolved in distilled water. Separately, concentrated stock solutions (100×, 500×, or 1000×) of amino acids, vitamins and para-aminobenzoic acid were prepared and added sequentially. Nutrients such as glucose or L-glutamine, and calcium D-pantothenate were omitted or added depending on the experimental design. After all components were fully dissolved, the pH was adjusted to 7.4. The media were then sterilized through a 0.2 μm PES membrane filter and stored at 4°C until use.

Sample preparation for metabolomics analysis

For intracellular nontargeted metabolomics, stimulated CD4^+^ T cells were seeded in 24-well plates with RPMI-1640 medium containing 10% FBS with same number of cells per sample. After 4 hours, the cells were quickly collected and washed with ice-cold PBS. Cellular metabolites were extracted using 80% methanol (stored at -80°C for at least 30 min). The metabolite extract was cleared by centrifugation at 16,000× *g* for 20 min at 4°C, followed by LC-HRMS analysis in polarity switching mode for nontargeted metabolomics.

For U-^13^C glucose (CLM-1396-5, Cambridge Isotope Laboratories, Andover, MA, USA) or ^13^C_5_, ^15^N_2_-glutamine (CNLM-1275-H-0.1, Cambridge Isotope Laboratories) tracing, stimulated CD4^+^ T cells were seeded in 24-well plates with U-^13^C glucose or ^13^C_5_, ^15^N_2_ glutamine-containing medium (glucose or glutamine-free RPMI-1640 medium supplemented 10% dialyzed FBS, and 2 g/L U-^13^C glucose or 2 mM ^13^C_5_, ^15^N_2_ glutamine) with same number of cells per sample. For pantothenate supplemented experiment, custom RPMI-1640 base media lacking glucose or glutamine, and pantothenate were used instead. For pantothenate-supplemented experiments, custom RPMI-1640 base media lacking glucose, glutamine, and pantothenate were used. Depending on the tracing condition, U-^13^C glucose (2 g/L) or ^13^C_5_, ^15^N_2_ glutamine (2 mM) was added back to the media supplemented 10% dialyzed FBS, with or without 2 μM pantothenate supplementation. After 4 hours, the cells were quickly collected and washed with ice-cold PBS. Cellular metabolites were extracted using 80% methanol (stored at -80°C for 30 min). The metabolite extract was cleared by centrifugation at 16,000× *g* for 20 min at 4°C, followed by LC-HRMS analysis in polarity switching mode for nontargeted metabolomics. From the leftover supernatant, 200 µl of the extract was removed, the solvent was evaporated via a Speedvac (EZ2elite, Genevac, Ipswich, UK), and the residue was reconstituted with 40 µl of 50% methanol. The reconstituted extract was centrifuged again at 16,000× *g* for 20 min at 4°C, and the supernatant was transferred to LC vials for liquid chromatography-mass spectrometry (LC-MS) analysis of palmitic acid and palmitoyl coenzyme A in negative mode.

For U-^13^C glucose tracing to ATP and CoA in CD4^+^ T cells from *Pdk1*^flox/flox^; *Cd4* Cre mice (for Fig. 1i, j and supplementary Fig. 4), the tracing process was conducted as described above. The cellular metabolites were extracted and derivatized with ice-cold extraction solvent (80% methanol:20% water containing 25 mM NEM and 10 mM ammonium formate, pH 7.0). After incubation on ice for 30 min, the NEM-derivatized metabolite extracts were cleared via centrifugation, and the supernatants were analyzed via LC‒HRMS in polarity switching mode. Peak area of [M+5] CoA is normalized by [M+5] ATP peak area to account for ATP fluctuations (for supplementary Fig. 4), ensuring that changes in [M+5] CoA reflect differences in CoA synthesis rather than ATP availability.

Sample preparation for lipidomics analysis

The cells were harvested and washed twice with ice-cold PBS, followed by extraction with ice-cold chloroform-methanol (1:2) containing 1 mg/ml butylhydroxytoluene (BHT). The samples were then sonicated for 6 cycles (30 seconds of sonication followed by 30 seconds of rest) in ice-cold water via a BMS Bioruptor. After a 30-minute incubation on ice, the samples were centrifuged at 16,000× *g* for 20 min, and the supernatant was transferred to glass vials for LC‒MS analysis.

Sample preparation for LC-QqQ-MS based glutamine quantification

For ^13^C_5_,^15^N_2_-glutamine tracing media samples, 40 µl of media was extracted with 960 µl of an extraction solvent containing unlabeled glutamine (G8540-25G, Sigma-Aldrich), achieving final concentrations of 100 µM for unlabeled glutamine. The samples were incubated on ice for 30 min and then centrifuged at 16,000× *g* for 20 min at 4°C. The supernatants were subsequently transferred to glass vials for LC-QqQ-MS analysis.

Sample preparation for lactate quantification

For U-^13^C-glucose tracing media samples, 40 µl of media was extracted with 960 µl of an extraction solvent. The samples were incubated on ice for 30 min and then centrifuged at 16,000× *g* for 20 min at 4°C. The supernatants were subsequently transferred to glass vials for LC‒HRMS in polarity switching mode.

References

1 Dibble, C. C. *et al.* PI3K drives the de novo synthesis of coenzyme A from vitamin B5. *Nature* **608**, 192-198 (2022).

2 Huang, L. *et al.* A family of metal-dependent phosphatases implicated in metabolite damage-control. *Nat Chem Biol* **12**, 621-627 (2016).

3 Kang, Y. P. *et al.* Non-canonical Glutamate-Cysteine Ligase Activity Protects against Ferroptosis. *Cell Metab* **33**, 174-189 e177 (2021).

4 Rampler, E. *et al.* Recurrent Topics in Mass Spectrometry-Based Metabolomics and Lipidomics-Standardization, Coverage, and Throughput. *Anal Chem* **93**, 519-545 (2021).


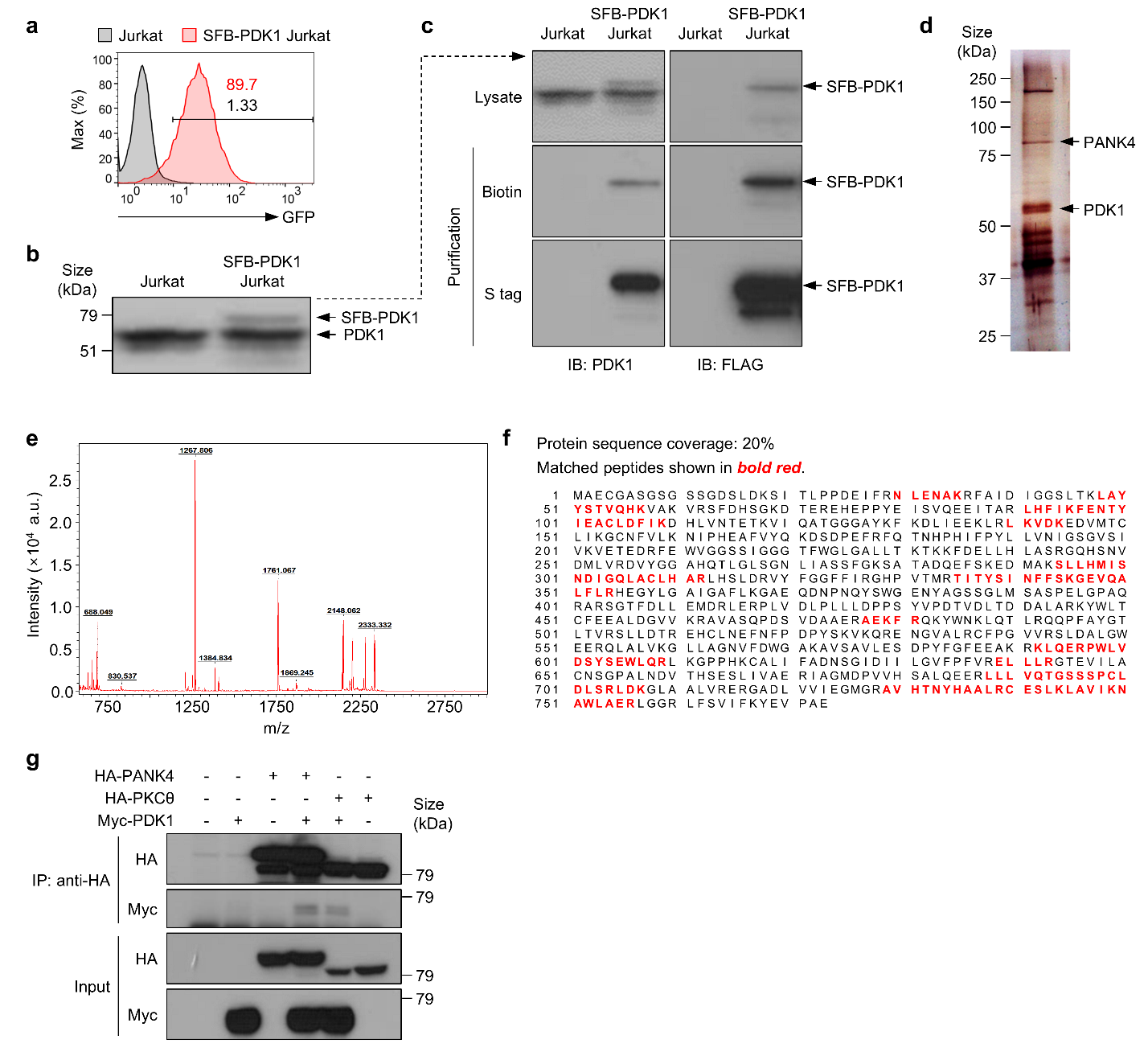


Figure. S1.

**Identification of PANK4 as a novel PDK1-binding protein.** **a** Flow cytometry of GFP^+^ cells. **b** Immunoblot analysis of lysates from Jurkat and SFB-PDK1 Jurkat cells (Jurkat cells expressing recombinant PDK1 with an S tag, a biotin tag, and a FLAG tag) using an anti-PDK1 antibody. This blot image is also included in (**c**). **c** Purification of recombinant PDK1 by biotin and S tag affinity pull-down. **d** Silver-stained gel image after recombinant PDK1 pull-down. **e**, **f** Mass fingerprinting analysis of copurified protein bands. **g** Coimmunoprecipitation assays using HEK293T cell lysates overexpressing the indicated proteins. Data for (**g**) are representative of two independent experiments. HA, hemagglutinin.


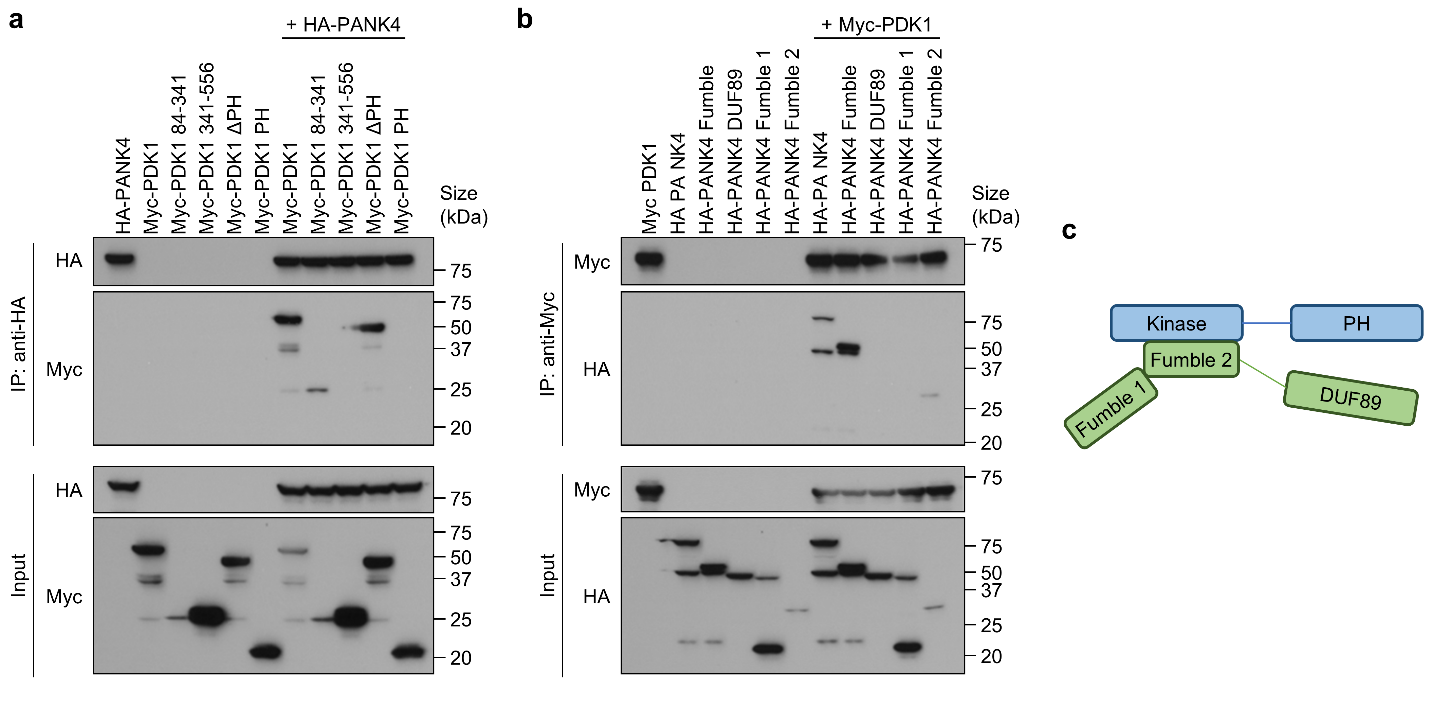


Figure. S2.

**Analysis of the interaction domain between PDK1 and PANK4.** **a**, **b** Coimmunoprecipitation assays using truncated forms of PDK1 (**a**) and PANK4 (**b**) expressed in HEK293T cells. **c** Schematic representation of the interaction between PDK1 and PANK4. HA, hemagglutinin.


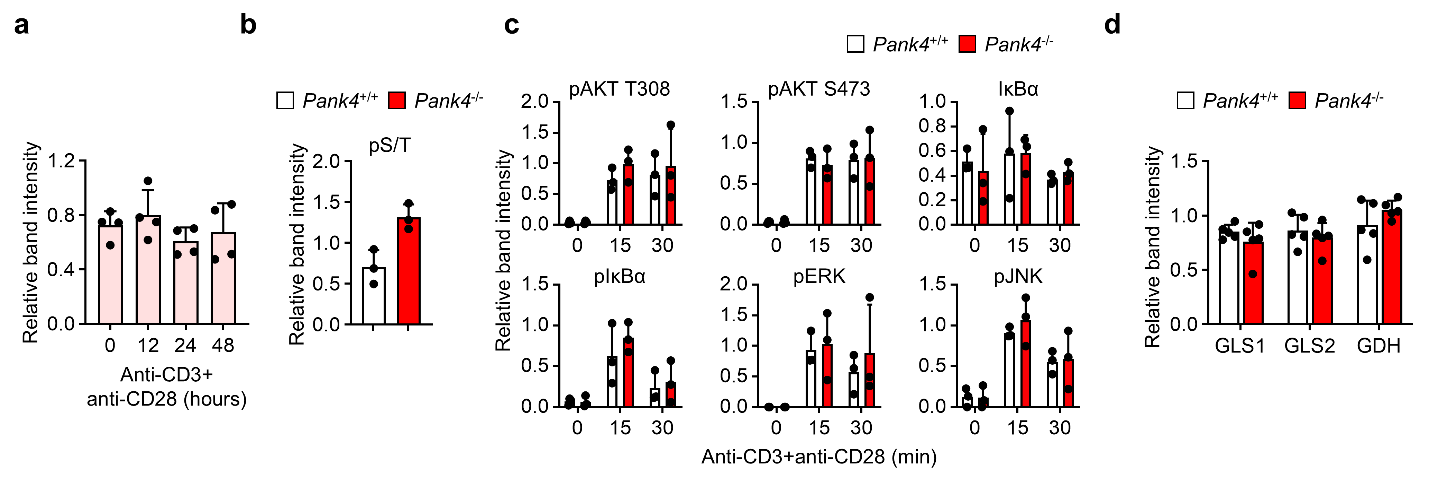


Figure. S3.

**Relative band intensities. a**-**d** Normalized band intensities of target protein corresponding to Fig. 1e (**a**), pS/T bands of Fig. 1f (**b**), Fig. 2d (**c**), and Fig. 5c (**d**)


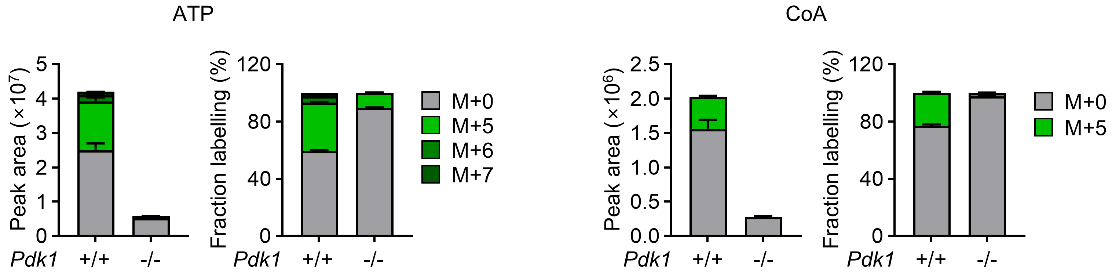


Figure. S4.

**U-^13^C-Glucose tracing of CoA and ATP.** Peak area and fraction labelling of ATP and CoA after 4 hours of U-^13^C-Glucose tracing of activated CD4^+^ T cells isolated from *Pdk1*^wt/wt^; *Cd4 Cre* (+/+) and *Pdk1*^flox/flox^; *Cd4 Cre* (-/-) mice.

**
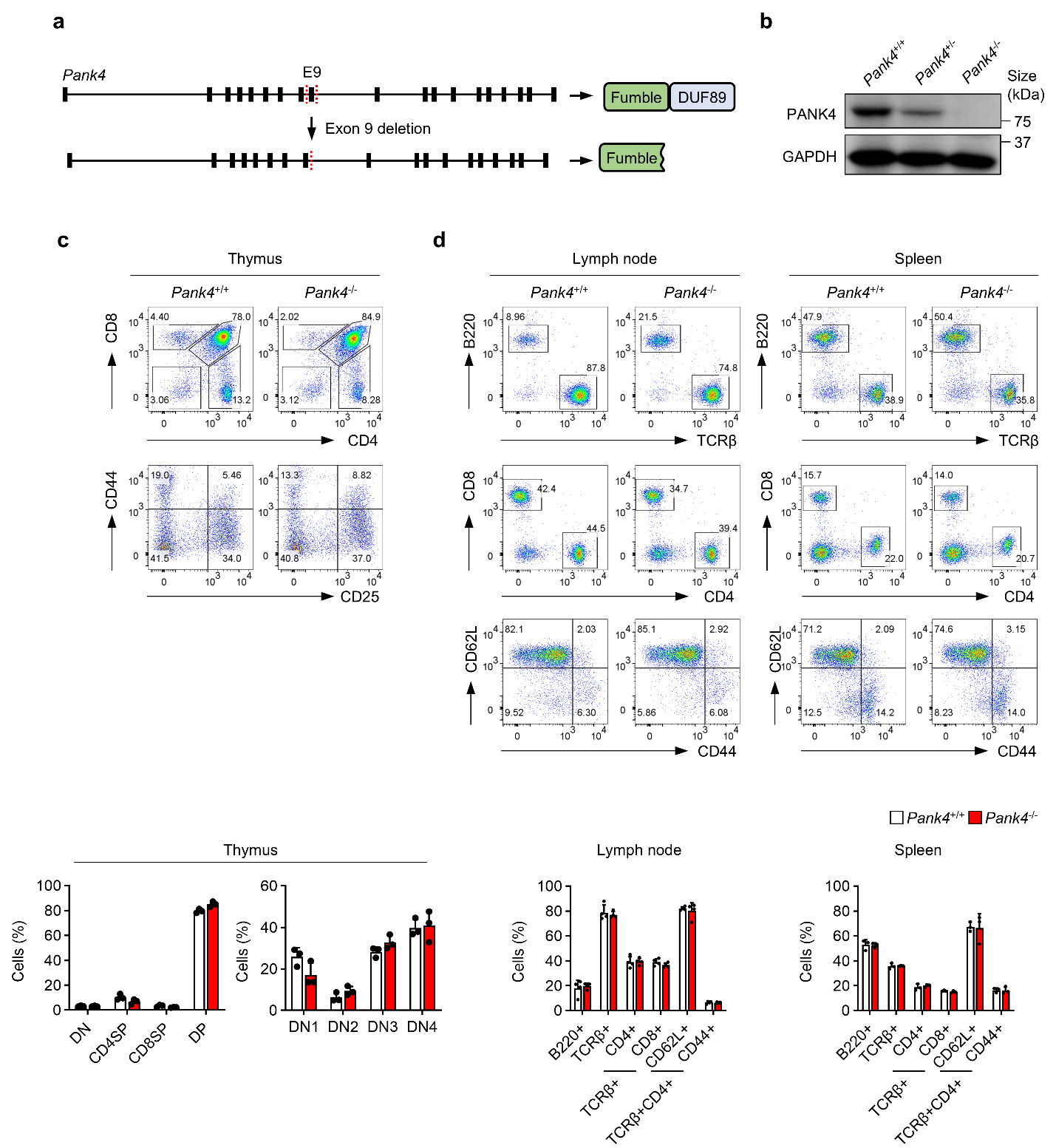
**

Figure. S5.

**Normal development of CD4^+^ T cells in *Pank4*^-/-^ mice.** **a** Schematic representation of the strategy for CRISPR-Cas9-mediated deletion of *Pank4*. **b** PANK4 expression in CD4^+^ T cells from *Pank4*^+/+^, *Pank4*^+/-^, and *Pank4*^-/-^ mice. **c** T cell population analysis in the thymuses of *Pank4*^+/+^ and *Pank4*^-/-^ mice (n = 3). **d** T cell and B cell population analysis of the lymph nodes and spleens of *Pank4*^+/+^ and *Pank4*^-/-^ mice (n = 4). Data are presented as the means ± SDs.


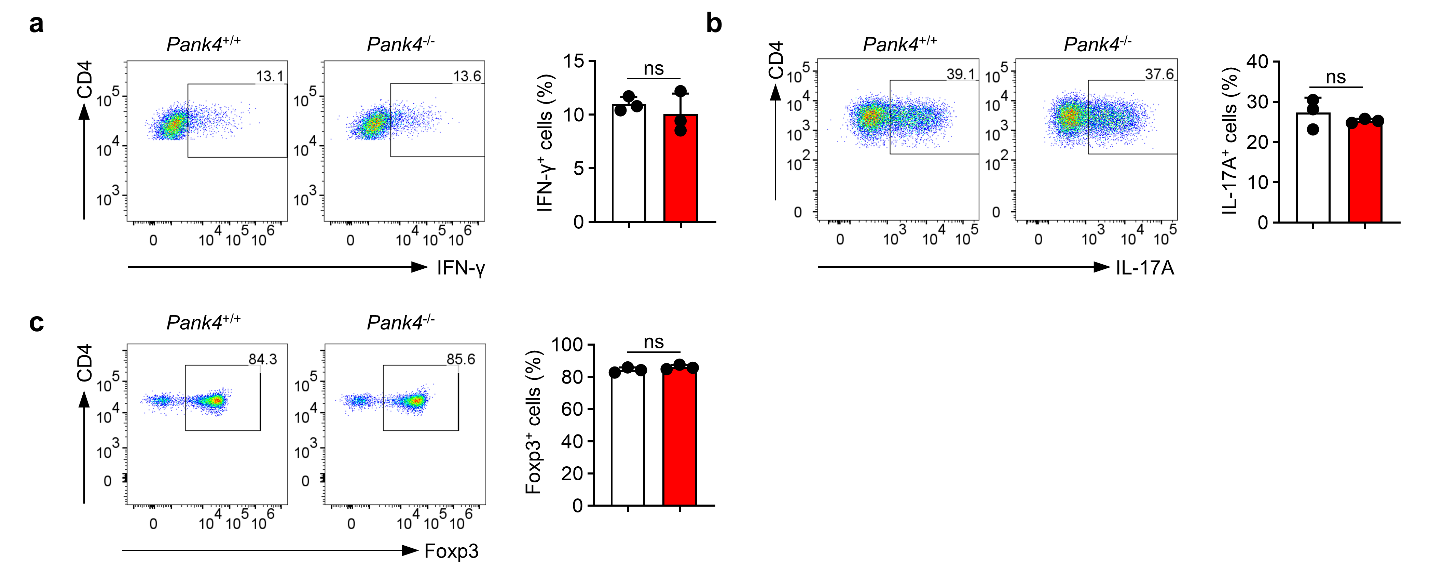


Figure. S6.

**Unaltered differentiation into T helper subsets *in vitro*.** **a**-**c** *In vitro* differentiation of *Pank4*^+/+^ and *Pank4*^-/-^ CD4^+^ T cells under Th1-polarizing conditions (**a**), Th17-polarizing conditions (**b**), and iTreg-polarizing conditions (**c**). The data are representative of three independent experiments and are presented as the means ± SDs. Statistical analysis was performed using an unpaired two-tailed t-test. ns, not significant; **P* ≤ 0.05, ***P* ≤ 0.01, and ****P* ≤ 0.001.


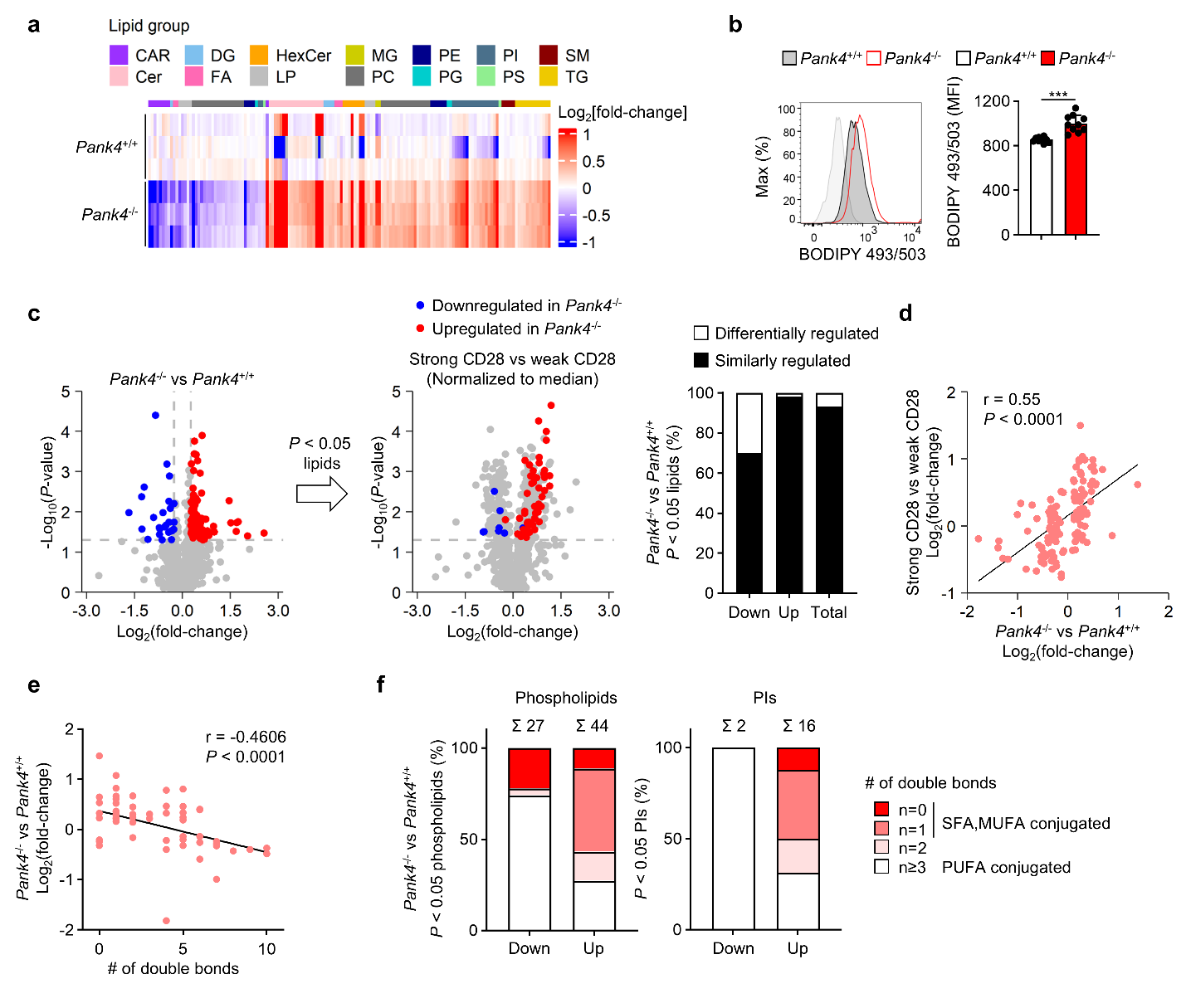


Figure. S7.

**Altered lipid profile of *Pank4*-deficient CD4^+^ T cells. a** Lipidomics of CD4^+^ T cells stimulated with anti-CD3 and anti-CD28 antibodies for 48 hours. **b** BODIPY 493/503 levels after stimulation of CD4^+^ T cells with anti-CD3 and anti-CD28 antibodies for 72 hours **c** Volcano plots representing significantly regulated lipids (P < 0.05) of *Pank4^-/-^* vs *Pank4^+/+^* cells in normal condition (left), and those strongly stimulated with CD28 (middle). Bar graph (right) summarizes the proportion of similarly and differentially regulated lipids in these two independent experiments. **d** Correlation analysis. Data are representative of two independent experiments. **e** Correlation analysis between the number of double bonds in all significantly regulated phospholipids in *Pank4*^-/-^ *vs.* wild-type CD4^+^ T cells in normal condition. **f** The percentage of phospholipids (left) and phosphatidylinositol (right) with different saturation levels among the significantly altered phospholipids/phosphatidylinositols (PIs) in *Pank4*^-/-^ compared with wild-type CD4^+^ T cells in normal condition. CAR, carnitine; Cer, ceramide; DG, diacylglycerol; HexCer, hexosylceramide; PC, phosphatidylcholine; PG, phosphatidylglycerol; PS, phosphatidylserine; ST, sterol; CE, cholesteryl ester; FA, fatty acid; LP, lysophospholipid; PE, phosphatidylethanolamine; PI, phosphatidylinositol; SM, sphingomyelin; TG, triacylglycerol.


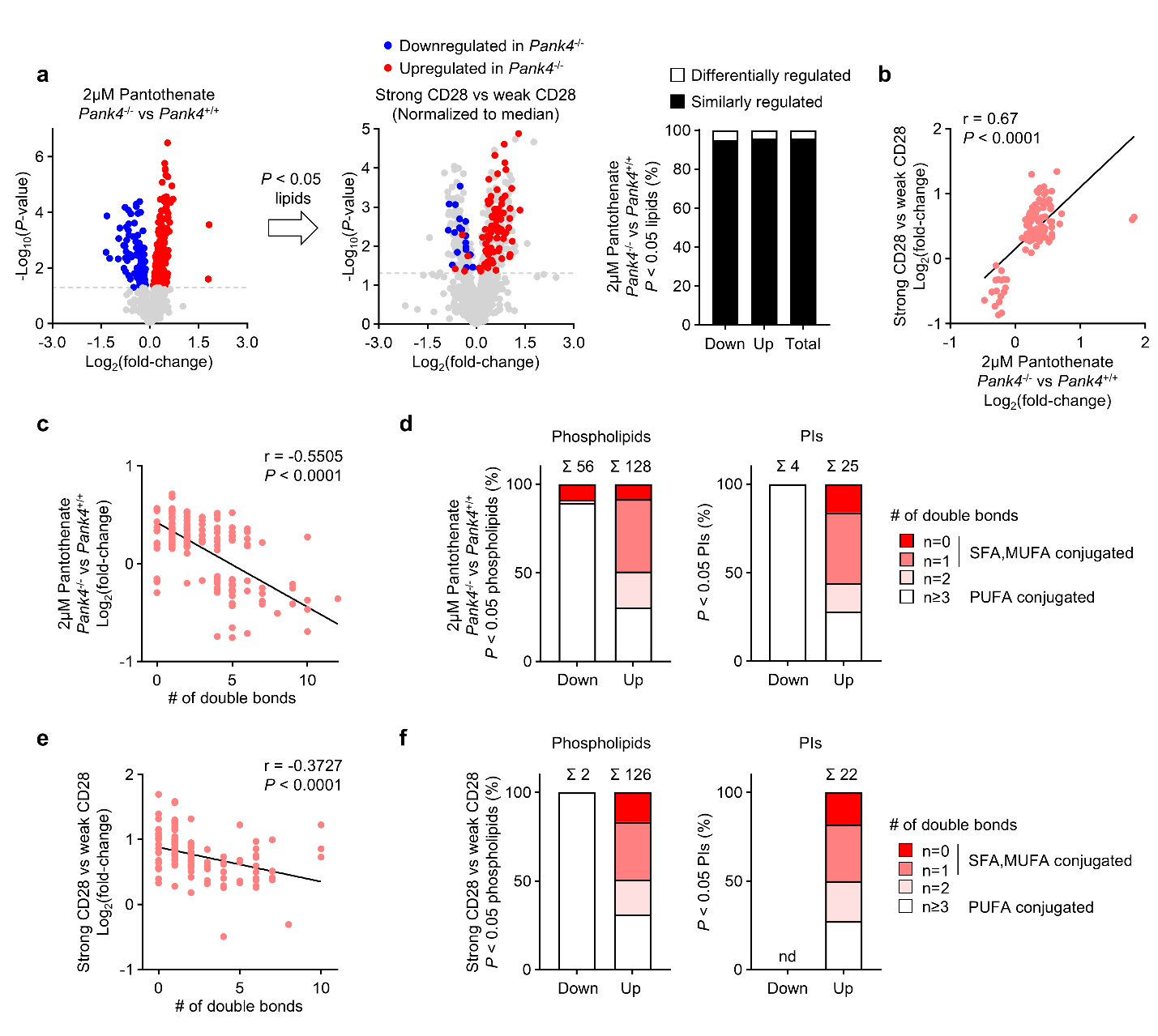


Figure. S8.

**Lipid profile of CD4^+^ T cells under pantothenate-supplemented and -depleted conditions. a** Volcano plot representing the common significantly regulated lipids between CD4^+^ T cells lacking PANK4 under pantothenate supplemented condition (left) and those strongly stimulated with CD28 (right). Lipids with *P* < 0.05 were selected from the left panel and plotted in the right panel. Bar graph summarizes the proportion of similarly and differentially regulated lipids in these two independent experiments. **b** Correlation analysis of lipids selected in (**a**). **c**, **e** Correlation analysis between the number of double bonds in all significantly regulated phospholipids in *Pank4*^-/-^ *vs.* wild-type CD4^+^ T cells under pantothenate supplemented condition (**c**) and CD4^+^ T cells strongly vs. weakly stimulated with CD28 (**e**). **d**, **f** The percentage of phospholipids (left) and phosphatidylinositol (right) with different saturation levels among the significantly altered phospholipids/phosphatidylinositols (PIs) in *Pank4*^-/-^ compared with wild-type CD4^+^ T cells under pantothenate supplemented condition (**d**) and in CD4^+^ T cells strongly vs. weakly stimulated with CD28 (**f**). Data are representative of two independent experiments.


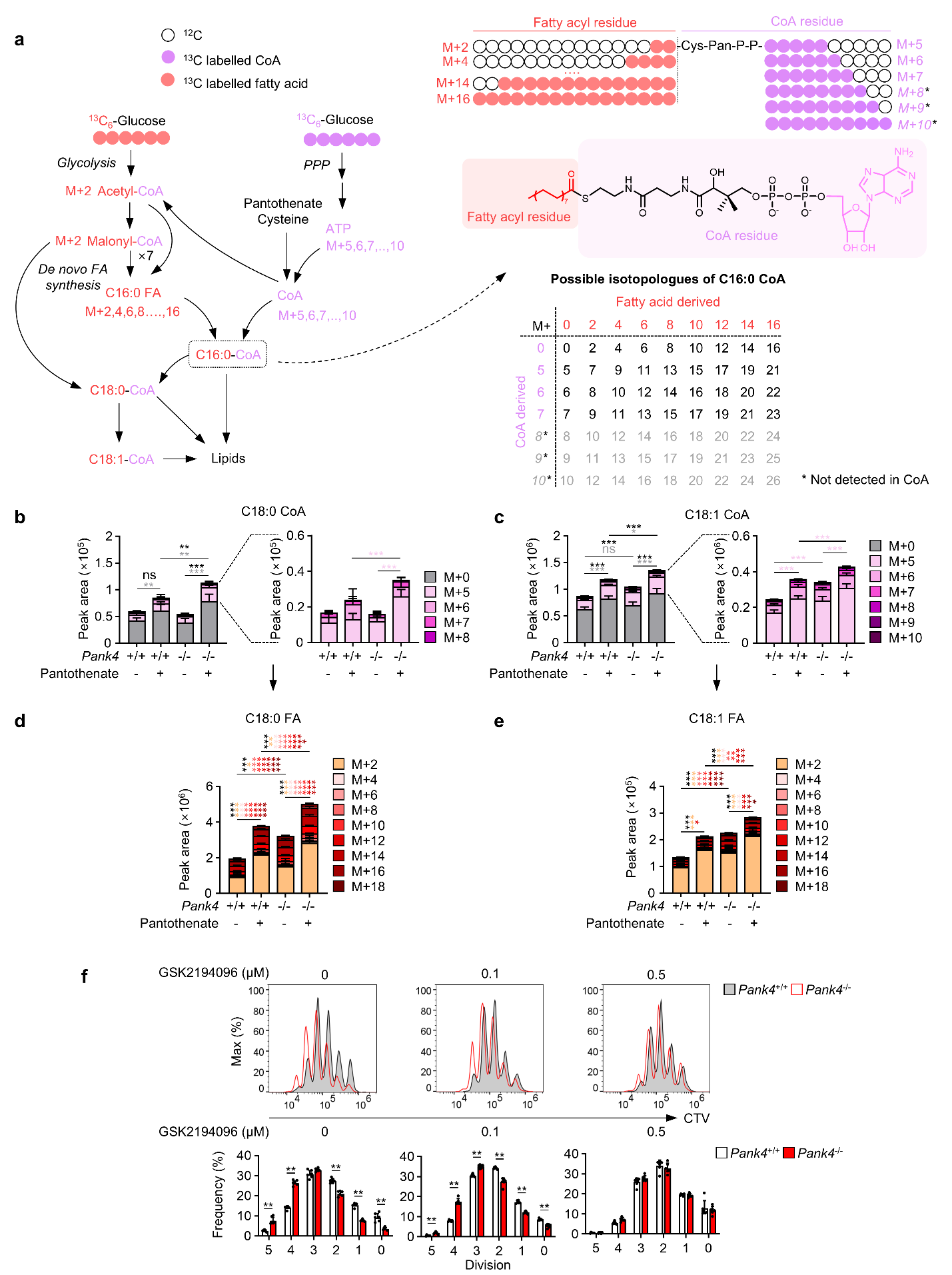


Figure. S9.

**Coenzyme A and fatty acid availability affects the lipid synthesis in CD4^+^ T cells. a** Schematics of ^13^C_6_ glucose tracing into fatty acid residue and coenzyme A residue of palmitoyl coenzyme A. **b**-**e** ^13^C_6_ glucose tracing of C18:0-CoA (**b**), C18:1-CoA (**c**), C18:0-FA (**d**) and C18:1-FA (**e**) after 4 hours in stimulated CD4^+^ T cells cultured with or without 2 μM pantothenate (n ≥ 7). **f** CellTrace violet proliferation assay in the presence of the indicated concentrations of GSK2194096. Data are representative of four independent experiments and are presented as the means ± SDs. Statistical analysis was performed using one-way ANOVA followed by Bonferroni post hoc test for (**b**-**e**) and an unpaired two-tailed t-test for (**f**). For (**b**-**e**), statistical analyses were performed on each labeled isotopologue (colored asterisks correspond to the respective isotopologue), and the sum of labeled isotopologues (indicated by black asterisks). ns, not significant, **P* ≤ 0.05, ***P* ≤ 0.01, and ****P* ≤ 0.001. C16:0-FA, palmitate; C16:0-CoA, palmitoyl CoA; C18:0-CoA, stearoyl CoA; C18:1- CoA, oleoyl CoA.


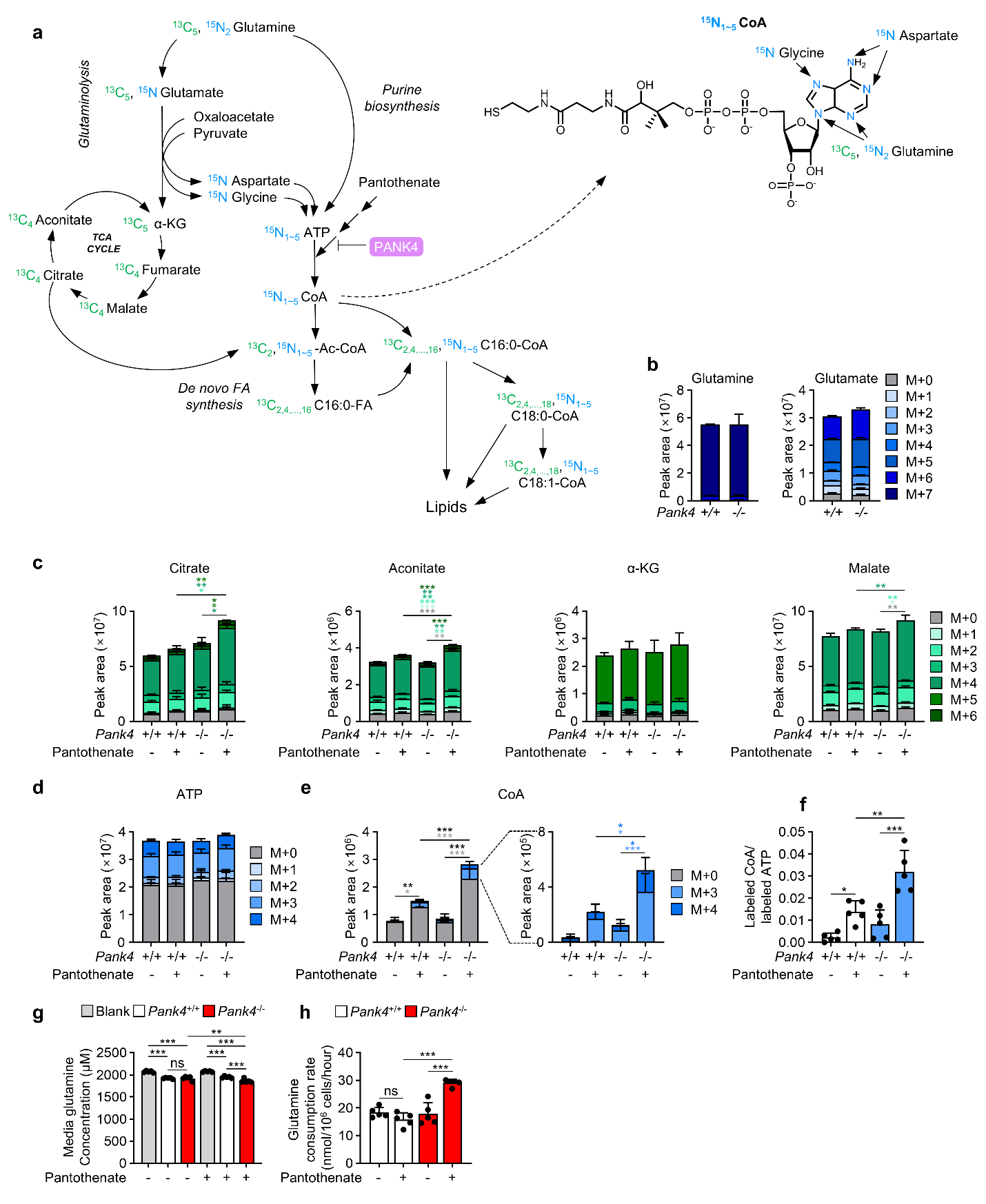


**Figure. S10.**

**Glutamine fuels TCA cycle and coenzyme A synthesis in CD4⁺ T cells under pantothenate regulation.** **a** Schematic illustrating how ¹³C₅,¹⁵N_2_-glutamine contributes both carbon to TCA-fatty acid synthesis and nitrogen to coenzyme A biosynthesis. **b**, **c** Levels and isotopic labeling of glutamine (**b**) and glutamate (**c**) after 4-hour tracing with ¹³C₅,¹⁵N_2_-glutamine in activated CD4⁺ T cells (n = 3). **c** ^13^C_5_, ^15^N_2_ glutamine tracing of TCA intermediates after 4 hours in stimulated CD4^+^ T cells cultured with or without 2 μM pantothenate (n = 5). **d**-**f** Quantification of ATP (**d**), CoA levels (**e**), and the peak area of labeled CoA normalized to labeled ATP (**f**) after 4-hour tracing using ¹³C₅,¹⁵N_2_-glutamine in activated CD4⁺ T cells under pantothenate-supplemented or -depleted conditions. **g** Residual ¹³C₅,¹⁵N_2_-glutamine concentration in culture media after 4-hour tracing under pantothenate-supplemented or -depleted conditions (n = 5). **h** Glutamine consumption rate of activated CD4^+^ T cells under pantothenate-supplemented or -depleted conditions (n = 5). Statistical analysis was performed using an unpaired two-tailed t-test for (**b**) and one-way ANOVA followed by Bonferroni post hoc test for (**c**-**h**). For (**b**-**e**), statistical analyses were performed on each labeled isotopologue (colored asterisks correspond to the respective isotopologue), and the sum of labeled isotopologues (indicated by black asterisks). ns, not significant, **P* ≤ 0.05, ***P* ≤ 0.01, and ****P* ≤ 0.001. α-KG, α-ketoglutarate; Ac-CoA, acetyl coenzyme A; C16:0-FA, palmitate; C16:0-CoA, palmitoyl CoA; C18:0-CoA, stearoyl CoA; C18:1- CoA, oleoyl CoA.


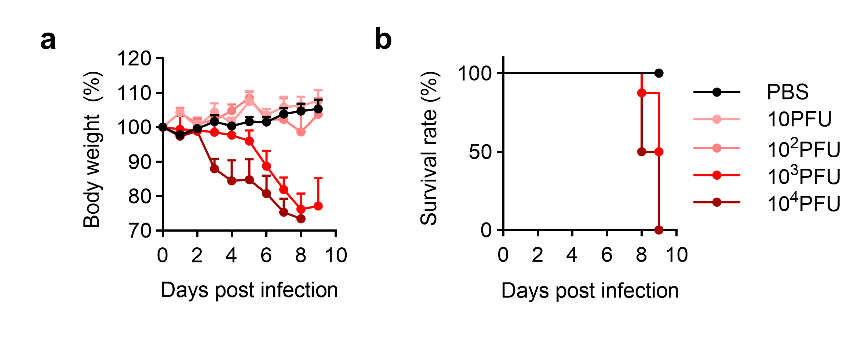


Figure. S11.

**Measurement of the murine lethal dose 50 (MLD_50_) for the A/Victoria/2570/2019 H1N1 virus.** 5-7 weeks old C57BL/6 mice were infected with 10 (n = 4), 10^2^ (n = 5), 10^3^ (n = 8) and 10^4^ (n = 4) plaque forming unit (PFU) of A/Victoria/2570/2019 virus. **a** Body weight curves. **b** survival curves.

Table S1.

**Resources.**

| RESOURCE | SOURCE | IDENTIFIER (Cat. No.) |
| --- | --- | --- |
| **Antibodies** | | |
| CD45R (B220) Monoclonal Antibody (RA3-6B2), APC-eFluor 780 | eBioscience | 47-0452-82 |
| CD11a (LFA-1alpha) Monoclonal Antibody (M17/4), FITC | eBioscience™ | 11-0111-81 |
| CD25 Monoclonal Antibody (PC61.5), PerCP-Cyanine5.5 | eBioscience | 45-0251-82 |
| BD Horizon PE-CF594 Hamster Anti-Mouse CD3e | BD | 562286 |
| CD3e Monoclonal Antibody (145-2C11), PerCP-Cyanine5.5 | eBioscience | 45-0031-82 |
| BD Horizon™ BUV395 Hamster Anti-Mouse CD3e | BD | 563565 |
| CD4 Monoclonal Antibody (GK1.5), eFluor 450 | eBioscience | 48-0041-82 |
| CD4 Monoclonal Antibody (GK1.5), APC | eBioscience | 17-0041-82 |
| BD Horizon BV421 Rat Anti-Mouse CD4 | BD | 562891 |
| CD4 Monoclonal Antibody (GK1.5), PE-Cyanine7 | eBioscience | 25-0041-81 |
| CD44 Monoclonal Antibody (IM7), APC | eBioscience | 17-0441-81 |
| CD45 Monoclonal Antibody (30-F11), PE-Texas Red | Invitrogen | MCD4517 |
| CD45.1 Monoclonal Antibody (A20), APC | eBioscience | 17-0453-81 |
| CD45.2 Monoclonal Antibody (104), PerCP-Cyanine5.5 | eBioscience | 45-0454-82 |
| BD Pharmingen™ PE Rat Anti-Mouse CD49d | BD | 557420 |
| CD62L (L-Selectin) Monoclonal Antibody (MEL-14), PerCP-Cyanine5.5 | eBioscience | 45-0621-82 |
| CD69 Monoclonal Antibody (H1.2F3) | eBioscience | 12-0691-82 |
| CD8a Monoclonal Antibody (53-6.7), PE | eBioscience | 12-0081-82 |
| FOXP3 Monoclonal Antibody (FJK-16s), Alexa Fluor 488 | eBioscience | 53-5773-82 |
| IL-2 Monoclonal Antibody (JES6-5H4), PE | eBioscience | 12-7021-82 |
| IFN gamma Monoclonal Antibody (XMG1.2), PerCP-Cyanine5.5 | eBioscience | 45-7311-82 |
| BD Pharmingen™ APC-Cy™7 Rat Anti-Mouse IFN-γ | BD | 561479 |
| BD Horizon BV605 Rat Anti-Mouse IL-10 | BD | 564082 |
| IL-17A Monoclonal Antibody (eBio17B7), PE | eBioscience | 12-7177-81 |
| TCR beta Monoclonal Antibody (H57-597) | eBioscience | 11-5961-82 |
| β-Actin Rabbit mAb | ABclonal | AC026 |
| Akt Antibody | CST | 9272 |
| Monoclonal Anti-Flag M2 antibody produced in mouse | Sigma-Aldrich | F3165 |
| GAPDH (H-12) antibody | Santa Cruz | sc-166574 |
| Glutamate Dehydrogenase 1/2 (D9F7P) Rabbit mAb | CST | 12793 |
| KGA/GAC Polyclonal antibody | Proteintech | 12855-1-AP |
| Anti-GLS2 antibody | GeneTex | GTX133243 |
| HA-Tag (C29F4) Rabbit mAb | CST | 3724 |
| Phospho-IκBα (Ser32/36) (5A5) Mouse mAb | CST | 9246 |
| Myc-Tag (9B11) Mouse mAb | CST | 2276 |
| Phospho-Akt (Ser473) (193H12) Rabbit mAb | CST | 4058 |
| Phospho-Akt (Thr308) (C31E5E) Rabbit mAb | CST | 2965 |
| PANK4 (D8N2C) Rabbit mAb | CST | 12055 |
| PANK4 (D6J4R) Rabbit mAb | CST | 12665 |
| PDPK1 (E-3) antibody | Santa Cruz | sc-17765 |
| Phospho-p44/42 MAPK (Erk1/2) (Thr202/Tyr204) (20G11) Rabbit mAb | CST | 4376 |
| Anti-IκB-α Antibody (H-4) | Santa Cruz | sc-1643 |
| Phospho-SAPK/JNK (Thr183/Tyr185) (81E11) Rabbit mAb | CST | 4668 |
| Pan Phospho-Serine/Threonine Rabbit pAb | ABclonal | AP0893 |
| **Mouse strains** | | |
| C57BL/6N-Pank4em1(IMPC)KMPC/KMPC | Korea Model Animal Priority Center | MOP1805002 |
| C57BL/6.SJL-Ptprca Pepcb/BoyJ | Jackson Laboratories | JAX:002014 |
| C57BL/6.129S7-Rag1tm1Mom/J | Jackson Laboratories | JAX:002216 |
| C57BL/6-*Pdk1*^flox/flox^; *Cd4-Cre* | [ref 19] | - |
| **virus strains** | | |
| A/Victoria/2570/2019-like virus (H1N1pdm09) | National Culture Collection for Pathogens (NCCP) | NCCP43400 |
| **Oligonucleotides** | | |
| Sequence | Target | |
| TGCGGCATGTTCTGGATTTG | Mouse *Il2* (F) | |
| TGGCACTCAAATGTGTTGTCAG | Mouse *Il2* (R) | |
| ACCAGAAGCGAATGGGAGTG | VI19 M gene (F) | |
| TCAGGCACTCCTTCCGTAGA | VI19 M gene (R) | |
| TGCAGCGGTTCAAATGATCCTCTCG | VI19 M gene (Probe) | |
| **Chemicals, peptides, and recombinant proteins** | | |
| [13C5, 15N2]-glutamine | Cambridge Isotope Laboratories | CNLM-1275-H-0.1 |
| [13C6]-glucose | Cambridge Isotope Laboratories | CLM-1396-5 |
| Acetonitrile (HPLC grade) | J.T.Baker | Mar-17 |
| CellTrace Violet Cell Proliferation Kit, for flow cytometry | Invitrogen | C34557 |
| D-Pantothenic acid hemicalcium salt / 100g | Sigma-Aldrich | P5155 |
| GSK2194069 | Sigma-Aldrich | SML1259 |
| H2O (LC-MS grade) | LiChrosolv | 1.15333.2500 |
| Isopropanol | J.T.Baker | I0356 |
| L-glutamine | Gibco | 25030081 |
| L-glutamine | Sigma-Aldrich | G8540-25G |
| MeOH (LC-MS grade) | LiChrosolv | 1.06035.2500 |
| N-ethylmaleimide (NEM) | Sigma-Aldrich | 04260-5G-F |
| PANK4 (Human) Recombinant Protein(P01) | Abnova | H00055229-P01 |
| PDK1 Protein, active | Merck | 14-452 |
| RPMI 1640 Medium w/L-Glutamine, w/o Pantothenic acid (Powder) | Usbio | R8999-01A |
| TVB2640 | Med Chem Express | HY-112829 |
| Ammonium formate | Sigma-Aldrich | 70221-25G-F |
| Ammonium acetate | Sigma-Aldrich | 73594-25G-F |
| Ammonium carbonate | Sigma-Aldrich | 379999-50G |
| Ammonium hydroxide | Sigma-Aldrich | 338818-5ML |
| BODIPY 493/503 | Invitrogen | D3922 |
| Protein G Sepharose 4 Fast Flow resin | Cytiva | 17061801 |
| Zombie Aqua™ Fixable Viability Kit | Biolegend | 423102 |
| **Critical commercial assays** | | |
| BD Cytometric Bead Array (CBA) Mouse Th1/Th2/Th17 CBA Kit | BD | 560485 |
| Malachite Green Phosphate Detection Kit | R&D Systems | DY996 |
| Silver stain kit | Pierce | 24612 |
| XFp Cell Mito Stress Test Kit | Agilent | 103010-100 |
| **Software** | | |
| GraphPad Prism | GraphPad | Version 10 |
| FlowJo | FlowJo | Version 10 |
| EL-Maven | https://resources.elucidata.io/elmaven | Version 0.12.0 |
| MS-DIAL | https://systemsomicslab.github.io/compms/msdial/main.html | Version 5.1.230912, 5.2.240218, or 5.5.241113 |
| Agilent MassHunter Qualitative Analysis | Agilent Technologies | Version B.08.00 |
