# Supplementary figures and images for: Pantothenate kinase 4 controls lipid synthesis for T-cell proliferation by modulating coenzyme A and glutaminolysis
Source: Signal Transduct Target Ther. 2025 Sep 18;10:302. doi: 10.1038/s41392-025-02385-7 (PMC12443970; doi:10.1038/s41392-025-02385-7)

**Fig. 1d**

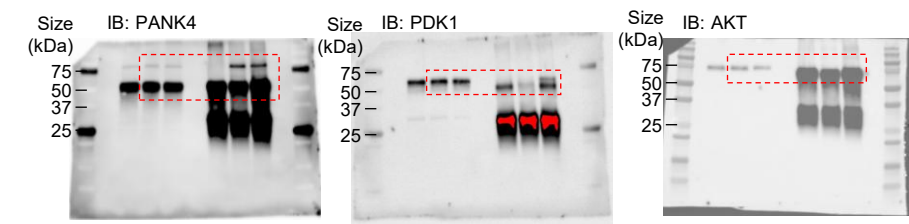

**Fig. 1e**

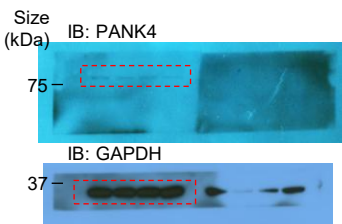

**Fig. 1f**

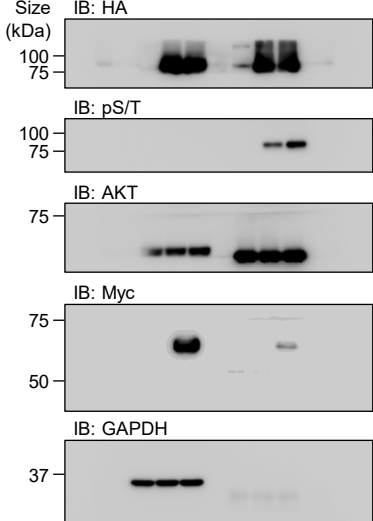

**Fig. 1g**

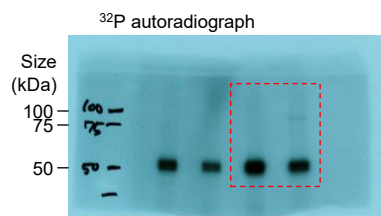

**Fig. 1h**

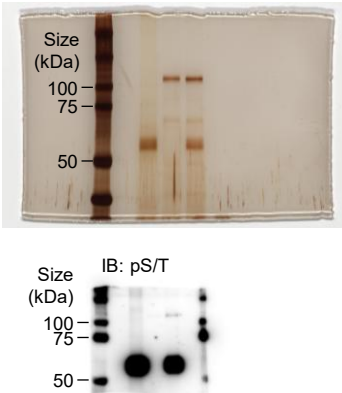

**Fig. 2d**

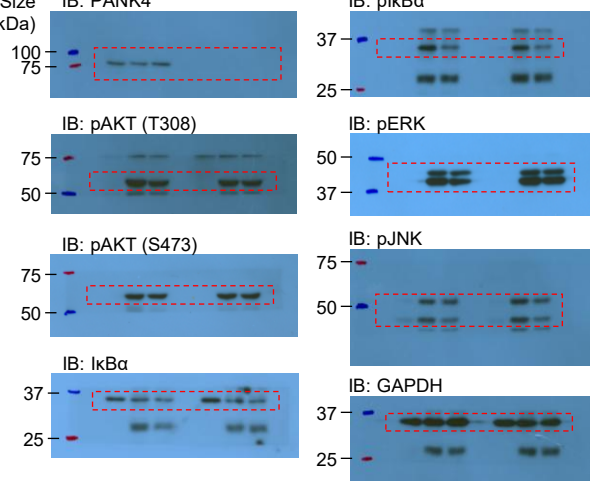

**Fig. 5d**

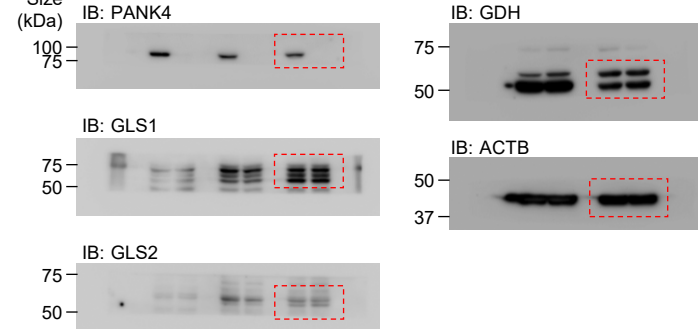

Fig. S1b, c

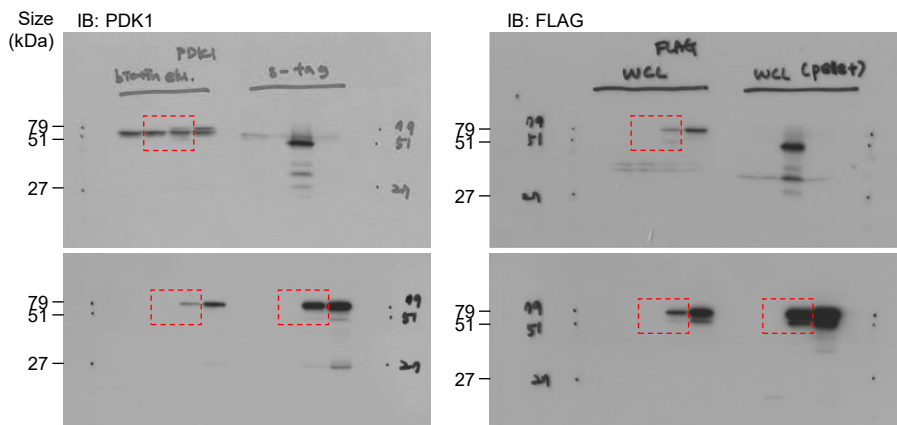

Fig. S1d

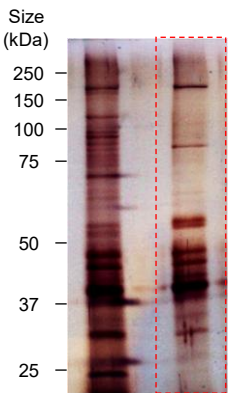

Fig. S1g

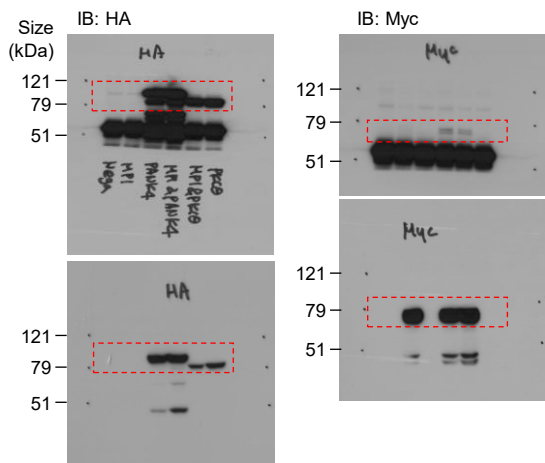

Fig. 2a, b

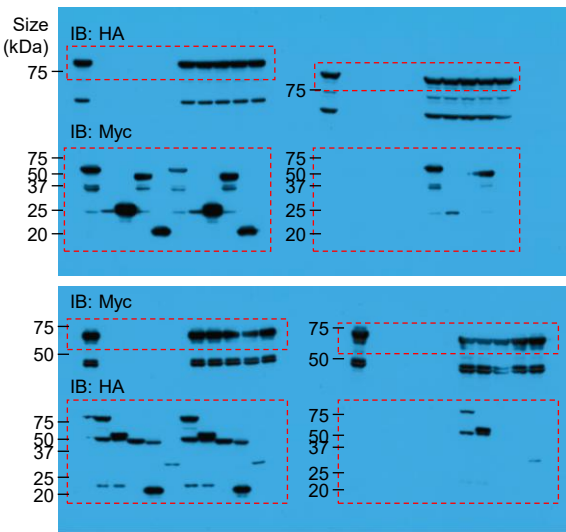

Fig. S5b

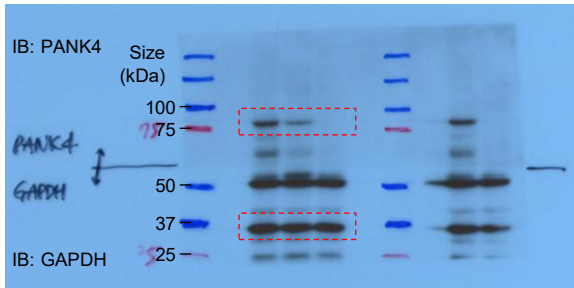

Supplement: Supplementary file 2 — Uncropped blot and gel [file 41392_2025_2385_MOESM2_ESM.pdf]

**Fig. 1a**

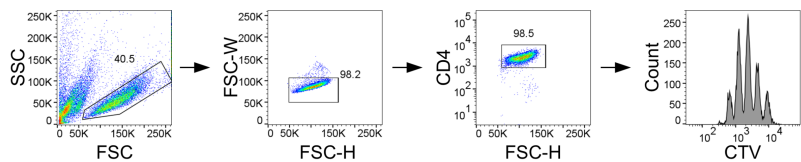

**Fig. 2a**

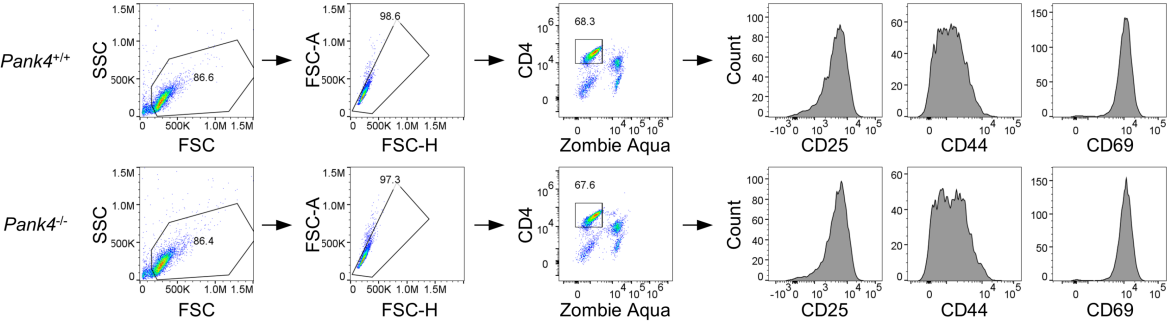

**Fig. 2c**

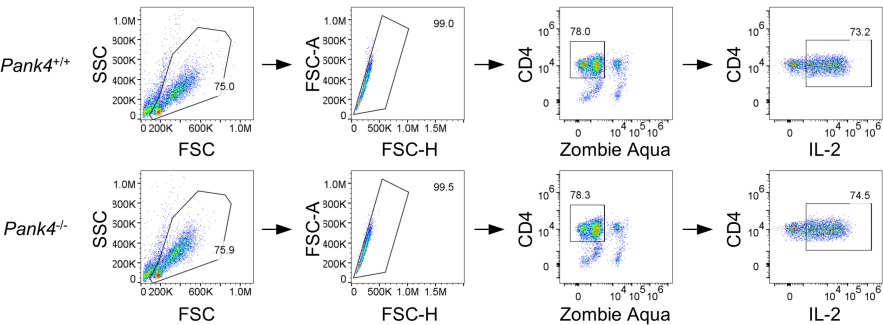

**Fig. 2e, 4h-j, 5k, S9f**

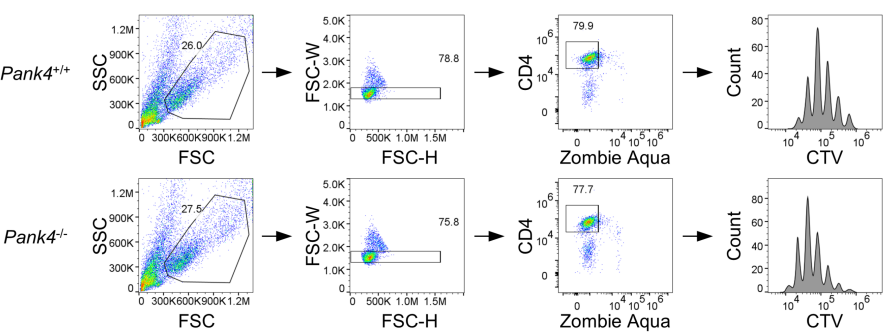

**Fig. 6e**

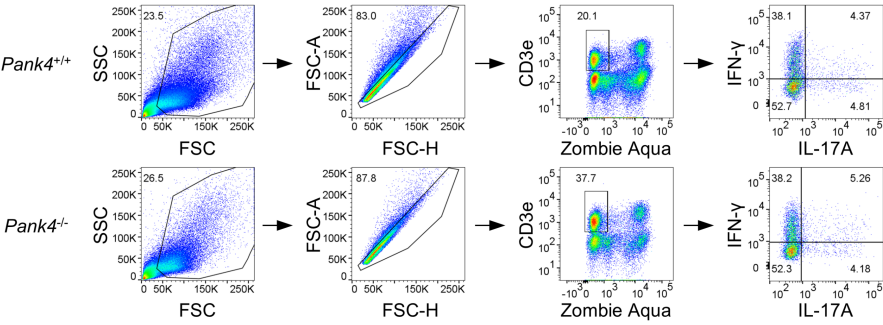

**Fig. 6i**

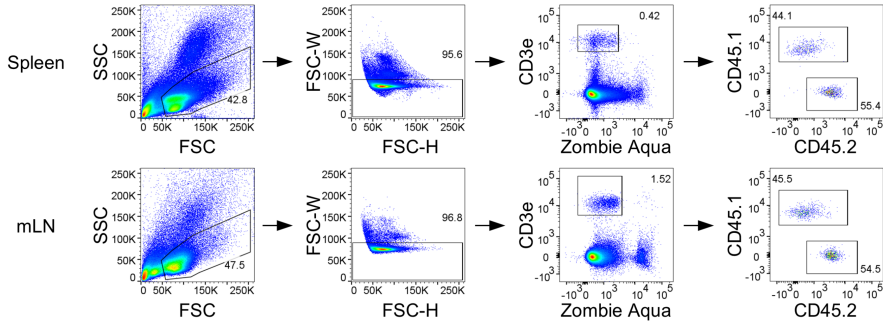

**Fig. 7h**

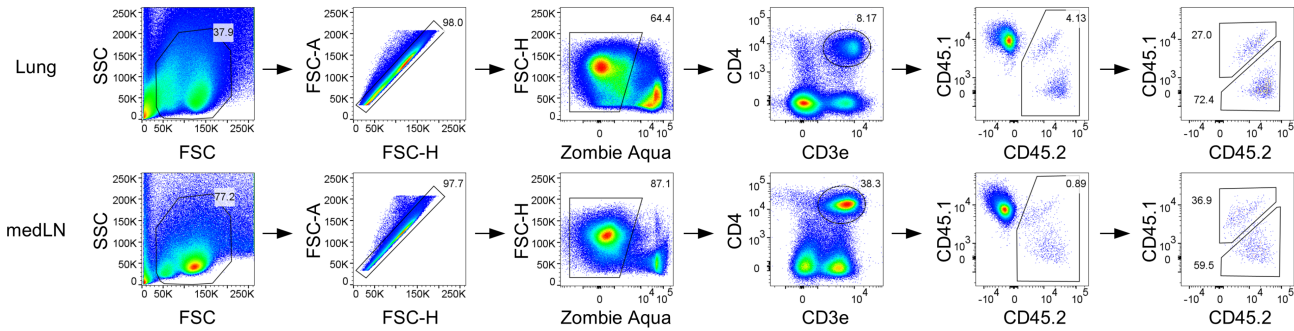

**Fig. S5c**

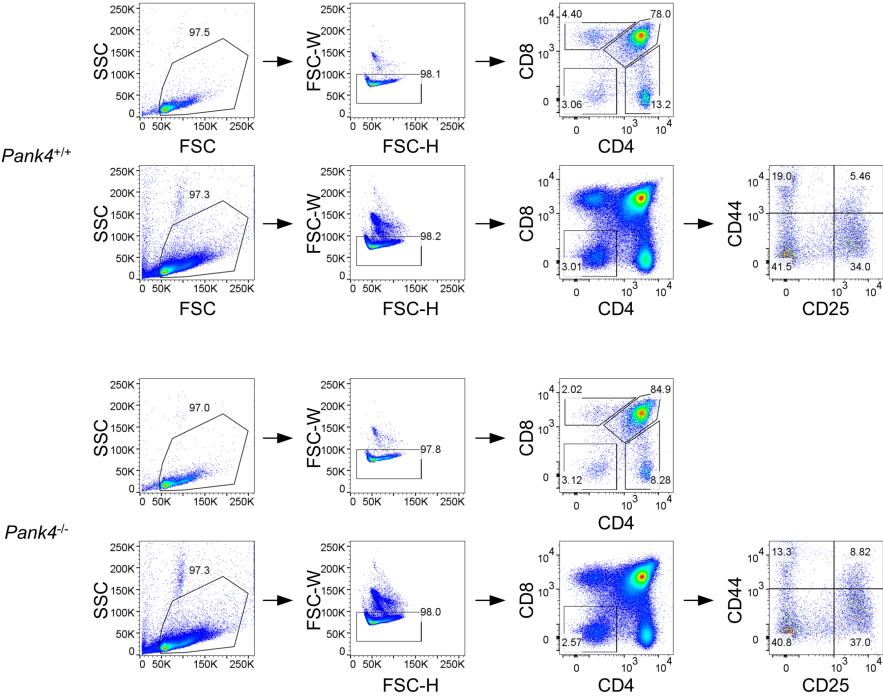

**Fig. S5d**

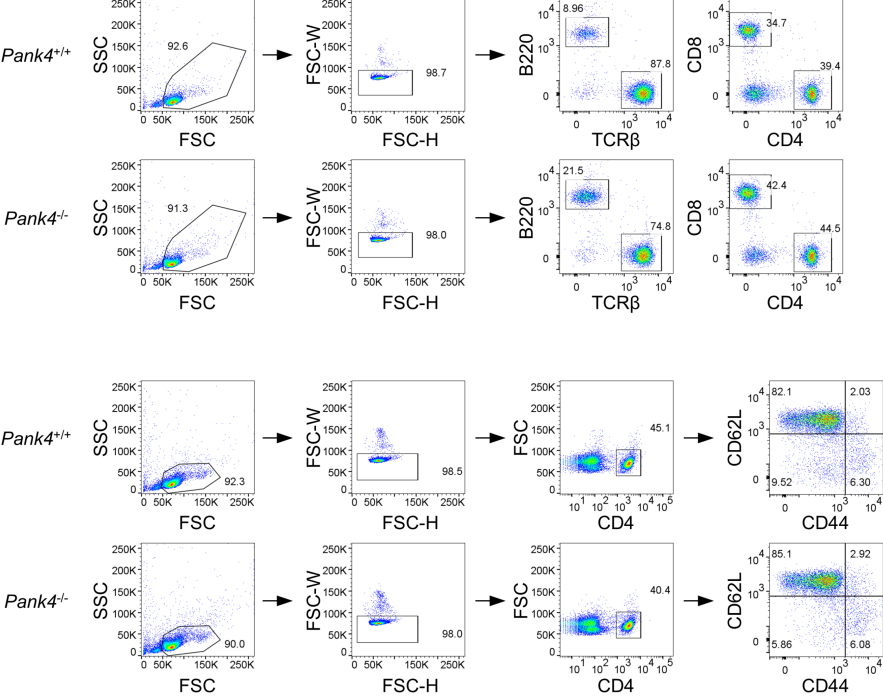

**Fig. S6a**

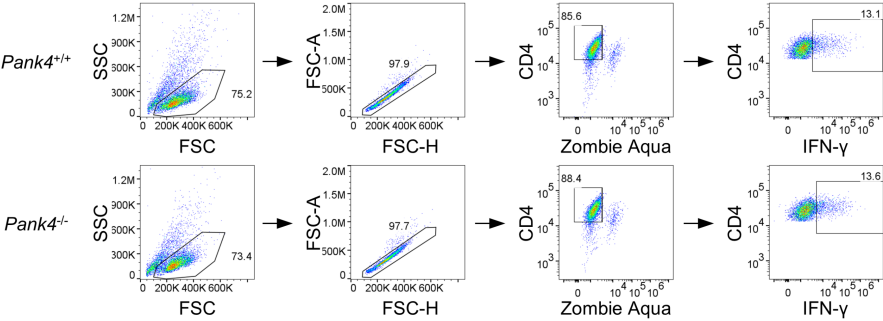

**Fig. S6b**

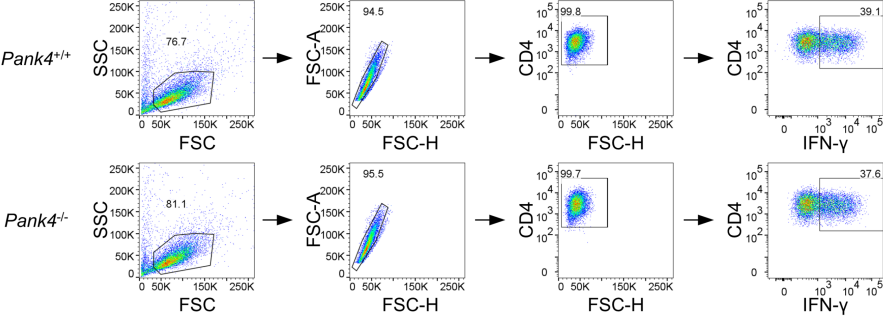

**Fig. S6c**

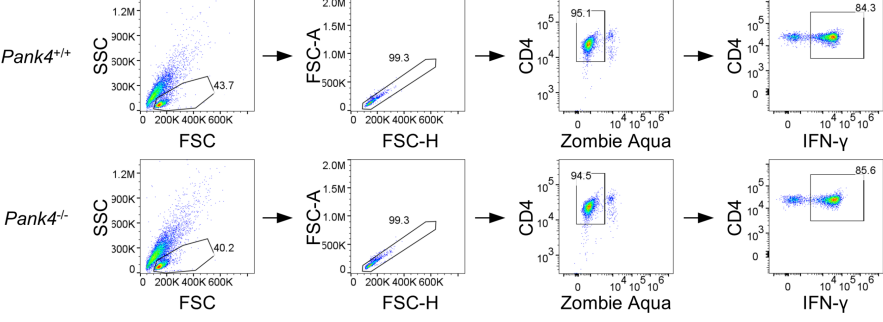

Supplement: Supplementary file 3 — Gating strategy [file 41392_2025_2385_MOESM3_ESM.pdf]
